# NELL-1: role and mechanisms in disease pathogenesis and development

**DOI:** 10.3389/fcell.2026.1783756

**Published:** 2026-04-22

**Authors:** Fang Mi, Qingrong Yue, Xi Deng, Lijun Jian, Yongkang Wu

**Affiliations:** 1 Nephrology and Blood Purification Unit, West China Hospital of Sichuan University Jintang Hospital, Jintang First People’s Hospital, Chengdu, China; 2 Department of Urology, West China Hospital of Sichuan University Jintang Hospital, Jintang First People’s Hospital, Chengdu, China; 3 West China Hospital of Sichuan University Jintang Hospital, Jintang First People’s Hospital, Chengdu, China; 4 Department of Laboratory Medicine, West China Hospital of Sichuan University, Chengdu, China

**Keywords:** NELL-1, bone regeneration, membranous nephropathy, tissue engineering, tumor suppressor

## Abstract

NELL-1 (NEL-like protein 1) is a multifunctional secreted glycoprotein that has been implicated in a variety of biological processes, including skeletal development, immune regulation, and tumorigenesis. Despite its significance, the regulatory mechanisms of NELL-1 across different tissues, the diversity of its signaling pathways, and its context-dependent roles in disease are not fully elucidated. This gap in understanding hinders the systematic development and clinical translation of potential applications. Current research suggests that NELL-1 influences skeletal development and regeneration through pathways such as Wnt/β-catenin, MAPK, and Runx3-Indian hedgehog (Ihh). Additionally, it plays crucial roles in conditions such as membranous nephropathy, various tumors, and neurodevelopmental disorders. The distinct isoforms of NELL-1, such as NELL-1_570_ and NELL-1-ΔE, exhibit specific functional properties and hold significant therapeutic promise, particularly in bone regeneration and tumor suppression. This review aims to provide a comprehensive analysis of the molecular mechanisms of NELL-1 in osteogenesis, chondrogenesis, immune regulation, and tumor suppression. It also explores various strategies to enhance its stability and targeting, including PEG modification and nanoparticle delivery systems. In conclusion, NELL-1 represents a key molecule with multi-tissue, multi-disease regulatory potential, and its value in both basic research and clinical translation is increasingly prominent. Future research should focus on its tissue-specific mechanisms, isoform functional optimization, and the development of safe and effective delivery systems to advance its applications in precision medicine.

## 1 Introduction

The development, homeostasis, and regeneration of the skeletal system rely on the precise regulation of growth factors. However, classic osteogenic factors such as bone morphogenetic proteins (BMPs) often cause side effects, including heterotopic ossification, due to their broad activity spectrum, which limits their clinical application (Shen et al., 2013). Consequently, there is an urgent need in the field of bone regeneration to discover novel osteogenic factors that combine high efficacy with target specificity.

In this context, NELL-1 (also known as NEL-like protein one or neural EGFL like 1), a secreted glycoprotein identified from a human fetal brain cDNA library in 1999, has attracted significant interest (Watanabe et al., 1996). Initial research established its crucial role in craniofacial skeletal development, where loss of function can lead to craniosynostosis (Zhang et al., 2002). Subsequent studies have continuously expanded the boundaries of its biological functions. Interestingly, NELL-1 precisely regulates osteogenesis and chondrogenesis *via* multiple signaling pathways such as Runx2/Ihh and Wnt/β-catenin, demonstrating higher bone specificity compared to BMP-2 (Zeng et al., 2022; James et al., 2015; Qin et al., 2016). However, its functional research has transcended the skeletal system in recent years. NELL-1 has been found to play roles in immune regulation, nervous system function, and tumor suppression in various malignant tumors (e.g., lung cancer, osteosarcoma, gastrointestinal tumors) (Zhai et al., 2019; Shen et al., 2015a; Gao et al., 2015). Notably, NELL-1 has been identified as a novel autoantigen in a subset of phospholipase A2 receptor (PLA2R)-negative membranous nephropathy, establishing its important position in the diagnosis and classification of autoimmune kidney diseases (specifically membranous nephropathy) (Sethi et al., 2020).

Despite its functional diversity, key challenges persist in understanding NELL-1, including its context-dependent molecular mechanisms, the functional differences among its isoforms, and the obstacles to its efficient and stable delivery as a therapeutic. This review aims to systematically outline the biological characteristics, expression pattern of NELL-1, and its role in various diseases. It also focuses on evaluating the latest clinical translation progress of NELL-1 as a diagnostic marker and therapeutic target, aiming to provide a solid theoretical framework and directional guidance to comprehensively understand its pleiotropic functions and promote its future applications in skeletal- and immune-related conditions, cancer, and other diseases.

## 2 Biological characteristics of NELL-1

### 2.1 Discovery and structural features of NELL-1

NELL-1 was initially isolated from a human fetal brain cDNA library and named NELL-1 (nel-like, type 1) due to its high homology with the chicken *nel* gene. The human gene *NELL-1* is located on chromosome 11p15.1-p15.2, spans approximately 906 kb, contains 21 exons, and encodes an 810-amino-acid protein. This protein contains six epidermal growth factor-like (EGF) repeat domains (Watanabe et al., 1996; Zhang et al., 2010).

NELL-1 is a secreted glycoprotein whose molecular structure is composed of three principal domains: an N-terminal thrombospondin (TSPN) domain, five von Willebrand factor type C (vWC) domains, and six epidermal growth factor-like (EGF) domains (Watanabe et al., 1996; Shen et al., 2015a; Zhang et al., 2010; Li et al., 2019; Kuroda et al., 1999). The five vWC domains are distributed as two N-terminal vWC domains following the CC region and three C-terminal vWC domains following the EGF repeats (Li et al., 2019; Kuroda et al., 1999). Sequence analysis has revealed that three of the six EGF domains possess calcium-binding capacity (Li et al., 2019; Zhao et al., 2018). Mutagenesis experiments have demonstrated that the heparin-binding activity of the TSPN domain is crucial for the interaction between NELL-1 and cell-surface proteoglycans (Takahashi et al., 2015). The C-terminal cysteine-rich region of the NELL-1 protein forms oligomers via intermolecular disulfide bonds. This oligomerization induces conformational changes in the protein, which is essential for NELL-1 to effectively mediate cell adhesion and spreading (Nakamura et al., 2014).

**FIGURE 1:**
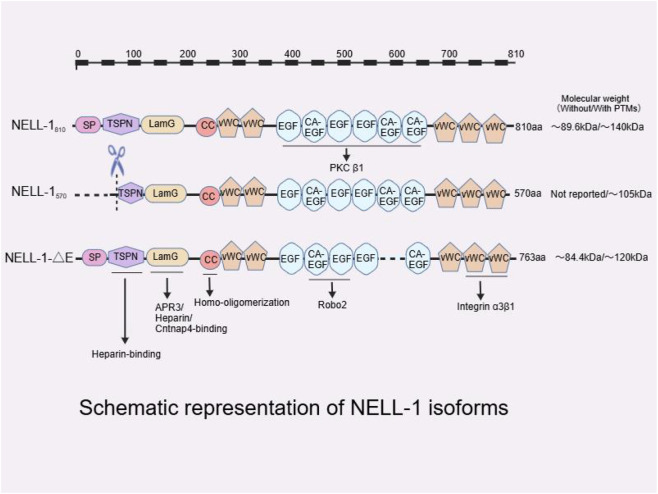
Schematic representation of NELL-1 isoforms. The full-length NELL-1_810_ (810 aa) contains an N-terminal signal peptide (SP), a thrombospondin N-terminal (TSPN) domain (with heparin-binding activity), a laminin G (LamG) domain (binding APR3, heparin, and Cntnap4), a coiled-coil (CC) region (responsible for homo-oligomerization), five von Willebrand factor C (vWC) domains (distributed as two N-terminal vWC domains following CC and three C-terminal vWC domains following the EGF repeats) (Watanabe et al., 1996; Shen et al., 2015a; Zhang et al., 2010; Li et al., 2019; Kuroda et al., 1999), and six epidermal growth factor-like (EGF) domains, three of which possess calcium-binding capacity “CA-EGF” (Li et al., 2019; Zhao et al., 2018). All EGF domains bind PKCβ1 (Li et al., 2019); the second and third EGF domains also bind Robo2 (Li et al., 2019); and the last two vWC domains bind integrin α3β1 (Li et al., 2019). Molecular weights are presented as theoretical value/glycosylated form (with PTMs): NELL-1_810_ 89.6 kDa/140 kDa ((Nakamura et al., 2014; Hasebe et al., 2012), NP_006148.2]); NELL-1-ΔE 84.4 kDa/120 kDa [(Zhao et al., 2018), NP_963845.1]); NELL-1_570_ (not reported/∼105 kDa) (Meyers et al., 2019; Pang et al., 2015). NELL-1_570_ is an N-terminally truncated splice variant lacking SP and part of TSPN (scissors indicate truncation site from alternative mRNA splicing, not proteolytic cleavage) (Meyers et al., 2019; Pang et al., 2015); NELL-1-ΔE lacks one calcium-binding EGF domain (Zhao et al., 2018). All isoforms are generated by alternative mRNA splicing. Domain architecture is based on published literature (Watanabe et al., 1996; Shen et al., 2015a; Zhang et al., 2010; Li et al., 2019; Kuroda et al., 1999). Created with BioGDP.com (Jiang et al., 2025).

Beyond the basic domain architecture, specific regions of NELL-1 have been shown to mediate distinct protein-protein interactions. The LamG domain binds APR3, heparin, and the receptor Cntnap4 (Li et al., 2019); the CC region mediates homo-oligomerization through disulfide bonds (Nakamura et al., 2014); all EGF domains interact with protein kinase C βI (PKCβ1), while the second and third EGF domains also bind Robo2 (Li et al., 2019); and the last two vWC domains (the fourth and fifth) serve as ligands for integrin α3β1 (Li et al., 2019). These functional interactions are schematically illustrated in Figure 1.

The NELL-1 gene is capable of producing multiple transcripts via alternative splicing mechanisms, yielding three primary isoforms that have been functionally characterized: (1) the full-length isoform NELL-1_810_; (2) an N-terminally truncated splice variant NELL-1_570_; and (3) a variant lacking a calcium-binding EGF-like domain NELL-1-ΔE (Zhao et al., 2018; Meyers et al., 2019; Pang et al., 2015).

NELL-1 is a highly glycosylated protein, with its monomeric subunit having a predicted molecular weight of approximately 90 kDa. However, through N-glycosylation, it matures into a glycoprotein with an approximate molecular weight of 140 kDa (Nakamura et al., 2014; Hasebe et al., 2012). These glycosylated subunits further assemble into a large, functional homotrimer with an apparent molecular weight exceeding 400 kDa, which is recognized as the bioactive form essential for the protein’s stability and biological activity (Nakamura et al., 2014; Hasebe et al., 2012).

### 2.2 NELL-1 isoforms and functions

Among the multiple isoforms, research has primarily elucidated the roles of the two major alternative splicing products: the full-length NELL-1_810_ and the N-terminally truncated NELL-1_570_. It is important to emphasize that this N-terminal truncation is determined at the mRNA splicing stage, not by proteolytic cleavage, resulting in their distinct expression patterns and functions (Meyers et al., 2019). Full-length NELL-1_810_ is highly expressed during embryogenesis, primarily participating in skeletal development; its expression level decreases significantly postnatally but does not completely disappear. In contrast, the N-terminally truncated NELL-1_570_ is expressed postnatally and can significantly promote the proliferation and osteogenic differentiation of various mesenchymal stem cells (MSCs) (Pang et al., 2015). Animal experiments further confirmed that local injection of the NELL-1_570_ protein can effectively repair calvarial defects, highlighting its potential as a novel strategy for bone regeneration therapy (Pang et al., 2015). The precise function of the low-level postnatal expression of full-length NELL-1_810_ remains unclear. A key scientific question is whether these two isoforms have distinct functions or regulate cellular processes by competing for the same cell surface receptors.

In addition to the two splicing isoforms, a transcriptional variant, NELL-1-ΔE, exhibits unique properties. The structural differences among these three isoforms are schematically illustrated in Figure 1. As a secreted protein, its intracellular localization (e.g., in the endoplasmic reticulum and Golgi apparatus) prior to secretion is similar to that of the full-length protein. Once secreted extracellularly, recombinant NELL-1-ΔE can bind to enolase-1 (ENO-1) localized on the cell membrane, thereby significantly inhibiting cell migration—an effect not observed with full-length NELL-1 (Zhao et al., 2018).

ENO-1 is a multifunctional enzyme that, in addition to its role in glycolysis, can localize to the cell surface where it acts as a plasminogen receptor (Díaz-Ramos et al., 2012). Binding of plasminogen to cell-surface ENO-1 promotes its conversion to plasmin, a protease that degrades extracellular matrix components and facilitates cell migration and invasion (Díaz-Ramos et al., 2012). By binding to ENO-1, NELL-1-ΔE may interfere with ENO-1-mediated plasminogen activation, thereby reducing extracellular matrix degradation and cell motility.

This mechanism has important implications for tumor biology, as the ability of NELL-1-ΔE to inhibit cell migration suggests a potential tumor-suppressive function, particularly in cancers where ENO-1 surface expression is upregulated and contributes to metastatic potential. Indeed, as discussed in Section 2.1.5, NELL-1-ΔE has been implicated in renal cell carcinoma, where its expression is associated with reduced cell migration and adhesion (Zhao et al., 2018).

This suggests that NELL-1-ΔE may exert potential tumor-suppressive functions by interfering with ENO-1-mediated glycolytic metabolism or signaling pathways, providing a new direction for fundamental cancer research and therapeutic development.

### 2.3 Expression and distribution

NELL-1 exhibits a spatiotemporally regulated expression pattern across multiple tissues, which underpins its diverse physiological and pathological roles. Its expression is dynamically regulated during development and varies between different cell types within organs.

#### 2.3.1 Systematic expression profile across tissues

NELL-1 expression is both tissue-specific and developmentally regulated, as detailed below for key organ systems.

##### 2.3.1.1 Developing skeleton

During embryogenesis, NELL-1 is highly expressed in osteochondral progenitor cells and differentiating osteoblasts, particularly within the cranial bones and growth plate cartilage, highlighting its critical role in skeletal patterning and mineralization (Watanabe et al., 1996; Pang et al., 2015). Postnatally, its expression in bone becomes more restricted but remains detectable in osteoblasts and periosteal cells involved in bone remodeling.

Beyond the skeleton, NELL-1 is also prominently expressed in the nervous system.

##### 2.3.1.2 Nervous system

NELL-1 is constitutively expressed in the central and peripheral nervous systems. High levels are found in specific brain regions such as the hippocampus, cortex, and cerebellum, as supported by data from the Human Protein Atlas (available from: https://v20.proteinatlas.org/ENSG00000165973-NELL1/tissue). Within these regions, expression localizes to neurons and certain glial cell populations (Karayannis et al., 2014; Li et al., 2018a). This distribution correlates with its functions in neural development, synaptic plasticity, and its association with neurological disorders.

In addition to its roles in skeletal and neural tissues, NELL-1 expression has been detected in several visceral organs, including the kidney.

##### 2.3.1.3 Kidney

In the kidney, NELL-1 expression is detected in podocytes and tubular epithelial cells. This expression is relevant to its newly identified role as a target antigen in a subset of membranous nephropathy (Sethi et al., 2020).

NELL-1 is also expressed in reproductive organs, although its function there remains to be elucidated.

##### 2.3.1.4 Reproductive system

Data from the Human Protein Atlas indicates that NELL-1 mRNA and protein are also highly expressed in the testis and prostate (https://v20.proteinatlas.org/ENSG00000165973-NELL1/tissue). While the specific functional role of NELL-1 in these tissues has not been experimentally established, its high expression raises the possibility of involvement in reproductive tissue physiology, a topic that warrants further investigation.

Finally, within the oral cavity, NELL-1 is present in the dental pulp-dentin complex.

##### 2.3.1.5 Dental pulp-dentin complex

Within human teeth, NELL-1 protein is primarily distributed in odontoblasts (cell bodies and processes), pulp fibroblasts, and vascular endothelial cells, indicating its role in dentinogenesis and pulp homeostasis (Liu et al., 2016; Han et al., 2019).

#### 2.3.2 Expression in pathological contexts: tumors and immune system

Tumor Tissues: Emerging evidence indicates that NELL-1 expression is dysregulated in various cancers, where it predominantly functions as a tumor suppressor. However, its role is context-dependent; both tumor-suppressive and, in certain malignancies, potential pro-tumorigenic effects have been reported. The mechanistic basis of this context-dependent behavior is discussed in Section 1.7, while its clinical relevance across different tumor types is reviewed in Section 2.1. Briefly, altered NELL-1 expression has been reported in osteosarcoma, glioblastoma, and other malignancies, influencing cell proliferation, invasion, and metastasis.

Immune System: While NELL-1 is not broadly expressed in classic hematopoietic immune cells, its role in immunomodulation is primarily indirect. It is produced by stromal and parenchymal cells (e.g., in bone or dental pulp) and signals to modulate the phenotype and function of resident or recruited immune cells, such as macrophages (Ni et al., 2024; Chen et al., 2025), as detailed in Section 1.5.4. As discussed in Section 1.7, this immunomodulatory capacity may extend to the tumor microenvironment, where NELL-1 could potentially influence anti-tumor immune responses. There is no strong evidence to date for its expression in lymphocytes, neutrophils, or other circulating immune cells under physiological conditions.

#### 2.3.3 Illustrative cases: NELL-1 in dental pulp and renal disease

The functional significance of its specific distribution is well-exemplified in two contexts:

Dental Pulp Repair: In rat and human dental pulp, NELL-1 co-localizes with neural markers (Substance P, nestin). *In vitro*, recombinant NELL-1 promotes the differentiation of dental pulp stem cells into neural-like cells, upregulating markers such as GFAP, nestin, and β-III tubulin, underscoring its role in pulp neural repair (Han et al., 2019).

Membranous Nephropathy (MN): Approximately 23% of PLA2R-negative MN patients exhibit specific deposition of NELL-1 antigen along the glomerular basement membrane, co-localizing with IgG. Circulating anti-NELL-1 antibodies are found in active disease, defining a distinct MN subtype and confirming NELL-1 as a pathogenic autoantigen expressed within the kidney (Sethi et al., 2020).

In summary, the tightly regulated, cell-type-specific expression of NELL-1 across developmental stages and in adult tissues—including brain, kidney, bone, reproductive organs, and teeth—directly determines its multifaceted functions in physiology and disease. Its dysregulation in cancer and its role as a stromal immunomodulator further expand the biological and clinical relevance of its expression pattern.

### 2.4 NELL-1 signaling pathways

NELL-1 exerts its diverse biological functions by interacting with multiple cell surface receptors. Two primary receptors have been identified: integrin β1 and Contactin-Associated Protein Like-4 (Cntnap4), which mediate distinct and complementary biological functions (James et al., 2015; Li et al., 2019; Li et al., 2018a). Integrin β1 primarily mediates NELL-1’s effects on cell adhesion and activates the canonical Wnt/β-catenin signaling pathway to promote osteoblast differentiation and inhibit osteoclast activity (James et al., 2015). In contrast, Cntnap4 serves as a high-affinity functional receptor critical for NELL-1’s osteogenic activity and potential neural functions, primarily through activation of MAPK/ERK signaling (Li et al., 2018a). This engagement activates conserved intracellular signaling hubs, including the Wnt/β-catenin, MAPK/ERK, and PI3K/Akt pathways, which converge to regulate key transcription factors (e.g., Runx2, Runx3, NFATc1) (Zhang et al., 2010; Nakamura et al., 2014). This core signaling network enables NELL-1 to precisely coordinate fundamental cellular processes like proliferation, differentiation, and survival. The following sections detail how this network is deployed and modulated within the specific contexts of chondrogenesis, osteogenesis (and its coupled angiogenesis), adipogenesis, and inflammation modulation.

Cntnap4, a member of the neurexin superfamily, has long been thought to function only in the nervous system, where it regulates GABA and dopamine release and is associated with autism spectrum disorders (Karayannis et al., 2014). However, subsequent research has identified Cntnap4 as a functional receptor for NELL-1 during osteogenesis, as it exhibits co-localization and high-affinity binding on the surface of osteoblasts. Moreover, Cntnap4 knockout completely abolished NELL-1-mediated activation of Wnt/MAPK signaling and osteogenic effects. Furthermore, a conditional Cntnap4 knockout mouse model recapitulated the cranial bone developmental defects observed in NELL-1-deficient mice. Notably, NELL-1 and Cntnap4 also co-localize on the surface of human hippocampal neurons, strongly suggesting that this ligand-receptor pair plays key roles not only in skeletal development but also in nervous system function (Li et al., 2018a).

#### 2.4.1 Chondrogenic mechanism

Research context: The role of NELL-1 in cartilage has been delineated using *in vitro* chondrogenic differentiation assays and *in vivo* genetic mouse models, relevant to both developmental skeletogenesis and postnatal repair processes.

Mechanistic insights: During chondrogenesis, the transcription factor Runx2 initiates cartilage formation by directly binding to the Ihh promoter (Zeng et al., 2022). However, evidence from a Runx2-deficient (Runx2^−/−^) mouse model revealed an alternative, Runx2-independent pathway: NELL-1 activates the transcription factor NFATc1, which directly binds to the Runx3 promoter, initiating the Runx3→Ihh signaling axis (Li et al., 2018b; Li et al., 2018c). Notably, although NELL-1 expression is regulated by Runx2, its pro-chondrogenic activity is functionally independent of Runx2 (Li et al., 2017). These findings indicate that NELL-1 is not merely a downstream effector but an autonomous key mediator promoting cartilage maturation.

Beyond its direct pro-chondrogenic effects, NELL-1 also exerts anti-inflammatory actions in cartilage by upregulating the transcription factor RUNX1, which suppresses NF-κB activity and subsequently inhibits the expression of pro-inflammatory cytokines such as IL-1β and downstream matrix-degrading enzymes including MMP-13 and ADAMTS-5 (Li et al., 2020).

Beyond its role in cartilage, NELL-1 also exerts potent effects on bone formation through distinct signaling pathways.

#### 2.4.2 Osteogenic and coupled angiogenic mechanism

NELL-1 is a central regulator of osteogenesis, with its deficiency linked to an impaired osteoblast-to-osteoclast ratio and age-related osteoporosis (James et al., 2015). The core mechanism involves NELL-1 binding to integrin β1, leading to activation of the Wnt/β-catenin pathway, which promotes osteoblast differentiation and inhibits osteoclast activity (James et al., 2015).

This osteogenic program is intricately fine-tuned by non-coding RNAs. Long non-coding RNAs (lncRNAs) mediate NELL-1’s effects by modulating Hedgehog and Wnt pathways (Xia et al., 2020), while NELL-1 upregulates circular RNAs circRFWD2 and circINO80 (Huang et al., 2019). These circRNAs act as molecular sponges, sequestering the osteogenic inhibitor hsa-miR-6817-5p and thereby revealing a novel “NELL-1 → circRNA → miRNA” regulatory axis in bone formation (Huang et al., 2019). Furthermore, NELL-1 ensures proper skeletal patterning and mineralization by regulating the Ihh-Parathyroid Hormone-related Protein (PTHrP) signaling feedback loop (Qi et al., 2019).

Coupling with Angiogenesis: Successful bone regeneration is contingent upon coupled angiogenesis. NELL-1 supports this essential process by synergistically enhancing the FGF2-AKT-eNOS pathway in vascular cells, thereby promoting the new blood vessel formation that is critical for bone graft vitality and healing (An et al., 2021).

Notably, the osteogenic effects of NELL-1 can be modulated by pharmacological agents targeting key metabolic regulators. For instance, combining NELL-1 with PPARγ inhibitors has been shown to enhance bone formation while reducing bone marrow adipogenesis, suggesting crosstalk between NELL-1 signaling and adipogenic pathways (James et al., 2015; Chermside-Scabbo et al., 2020).

In addition to promoting osteochondrogenesis, NELL-1 influences mesenchymal stem cell fate determination by modulating adipogenesis.

#### 2.4.3 Adipogenic mechanism

Consistent with its depiction in the graphical abstract (Figure 1), NELL-1 also influences mesenchymal stem cell fate determination by modulating adipogenesis. Evidence suggests that NELL-1 signaling can inhibit adipogenic differentiation, thereby promoting a shift in stem cell commitment toward the osteochondral lineage (James et al., 2015; Pang et al., 2015). This inhibitory effect on fat formation further underscores its primary role as a pro-osteogenic factor and highlights its potential relevance in metabolic bone disorders.

NELL-1 also plays a key role in regulating inflammation, complementing its functions in skeletal tissue formation.

#### 2.4.4 Anti-inflammatory mechanism

NELL-1 plays a key role in regulating inflammation, modulating macrophage polarization and related signaling pathways. In addition to its effects on macrophages, NELL-1 directly suppresses pro-inflammatory cytokines including IL-1β and TNF-α in various cell types, contributing to its tissue-protective effects in osteoarthritis and other inflammatory conditions (Wang et al., 2018). In periodontal inflammation, NELL-1 promotes macrophage polarization toward the anti-inflammatory M2 phenotype by activating the JNK/MAPK pathway, reducing tissue destruction (Ni et al., 2024). In dental pulp inflammation, however, NELL-1 exerts its anti-inflammatory effect primarily *via* the p38/ERK MAPK pathway, with the JNK pathway playing no significant role, indicating pathway specificity (Cao et al., 2021).

Furthermore, NELL-1 has been confirmed to significantly inhibit lipopolysaccharide (LPS)-induced M1 macrophage activation. Specifically, NELL-1 downregulates the expression of M1 markers (CD86) and pro-inflammatory cytokines (TNF-α, IL-6, IL-1β), while upregulating M2 markers (VEGF, Arg-1, CD206), thereby reversing the M1 polarization trend. This research further proposes that NELL-1’s anti-inflammatory effect may rely on the regulation of the NF-κB pathway (Chen et al., 2025). This aligns with findings that NELL-1 alleviates BMP2-induced inflammatory responses, a mechanism also associated with NF-κB pathway inhibition (Shen et al., 2013).

Collectively, the above studies substantiate that NELL-1 regulates macrophage polarization and inflammatory cytokine expression through various signaling pathways (e.g., MAPK and NF-κB), demonstrating broad anti-inflammatory potential in different inflammatory models.

#### 2.4.5 Beyond macrophages: potential roles in adaptive and innate immunity

While the anti-inflammatory role of NELL-1 is most characterized in macrophages, its potential impact on other immune cells remains an important area for future investigation. Given its expression in lymphoid tissues and modulatory effects on key pathways (e.g., NF-κB, MAPK) that are central to lymphocyte activation, it is plausible that NELL-1 may influence T cell or B cell function. Furthermore, its role in neutrophil or natural killer (NK) cell biology has not been explored. A comprehensive understanding of NELL-1’s immunomodulatory capacity is particularly crucial in the context of cancer. The tumor microenvironment is a complex ecosystem involving macrophages, lymphocytes, and other immune cells. As discussed in Section 2.1, dysregulation of NELL-1 in tumors may contribute to immune evasion or modulation not only through macrophages but potentially *via* these other, yet-to-be-defined immune interactions. Therefore, expanding research on NELL-1’s effects across the full spectrum of immune cells will be essential to fully elucidate its role in both physiological inflammation and cancer immunology.

#### 2.4.6 NELL-1 as an autoantigen: implications for immune tolerance breakdown

The relationship between NELL-1 and the immune system extends beyond immunomodulation to a direct pathogenic role in antibody-mediated autoimmunity. This is most clearly exemplified in NELL-1-associated membranous nephropathy (MN). Under physiological conditions, immune tolerance prevents attack against self-proteins like NELL-1. However, in susceptible individuals, a breakdown of this tolerance—potentially due to genetic predisposition, environmental triggers, or molecular mimicry—leads to the production of pathogenic IgG autoantibodies against NELL-1. These antibodies bind to NELL-1 antigen expressed on glomerular podocytes, forming *in situ* immune complexes within the glomerular basement membrane. This deposition triggers the classical complement cascade (e.g., forming the C5b-9 membrane attack complex), resulting in podocyte injury, proteinuria, and the clinical manifestations of MN (Sethi et al., 2020; Dantas et al., 2023).

This paradigm critically positions NELL-1 not only as a paracrine/autocrine signaling molecule but also as a key target antigen whose recognition by the adaptive immune system directly drives organ-specific autoimmune pathology, as detailed in Section 2.2.1.

#### 2.4.7 Complexity of NELL-1 regulation

The NELL-1 signaling network is itself subject to complex regulation, highlighting its integration into broader physiological contexts. For instance, miR-27a upregulates NELL-1 expression and inhibits the JNK/Wnt/β-catenin pathway, protecting human mitral valve interstitial cells from inflammatory damage (Chen et al., 2018). Separately, the growth factor BMP9 participates in bone metabolism regulation by modulating the osteoblast-osteoclast balance and NELL-1 expression (Song et al., 2020). These examples illustrate the multi-layered control of NELL-1 activity.

**FIGURE 2:**
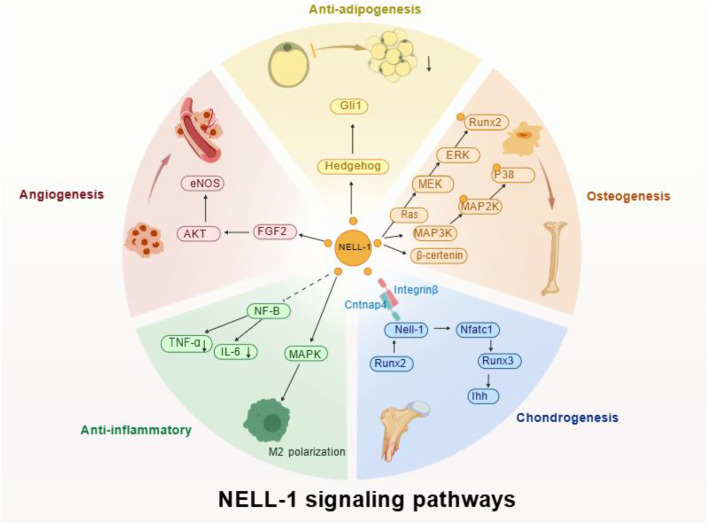
NELL-1 Signaling Pathways: NELL-1 can bind to cell surface receptors Cntnap4 and integrin β1. In osteogenesis, it activates Wnt/β-catenin and ERK/MAPK; NELL-1 promotes chondrogenesis by activating the transcription factor Nfatc1, initiating the Runx3→Ihh signaling axis; NELL-1 regulates macrophage polarization and inflammatory cytokine expression via MAPK and NF-κB pathways; NELL-1 significantly enhances the FGF2-AKT-eNOS pathway, promoting angiogenesis; NELL-1 inhibits adipogenesis via the Hedgehog signaling pathway. Created with BioGDP.com (Jiang et al., 2025).

Overall, NELL-1 plays a central regulatory role in osteogenesis, chondrogenesis, angiogenesis, adipogenesis, and inflammatory regulation through multiple signaling pathways, including Wnt/β-catenin, MAPK, Ihh, FGF2-AKT-eNOS, and Hedgehog (Figure 2).

The diverse biological functions resulting from these signaling pathways, including osteogenesis, chondrogenesis, adipogenesis, angiogenesis, anti-inflammation, cranial development, and dental tissue regeneration, are discussed in detail in Section 3.2.

### 2.5 Glycosylation and its impact on NELL-1 therapeutic potential

The glycosylation pattern of NELL-1 is not merely a structural feature but a critical determinant of its therapeutic potential. The extensive N-glycosylation is essential for proper protein folding, oligomerization, and protection from proteolytic degradation, thereby influencing its half-life and bioavailability (Gupta and Shukla, 2018). The mature, glycosylated homotrimer represents the bioactive form of NELL-1, capable of interacting with cell surface receptors like integrin β1 to initiate downstream signaling (James et al., 2015; Nakamura et al., 2014).

An important consideration for the clinical translation of NELL-1-based therapies is that the glycosylation pattern can vary depending on the expression system used for recombinant protein production. NELL-1 produced in bacterial systems (e.g., *E. coli*) lacks glycosylation entirely, which may result in a protein that is improperly folded, insoluble, or biologically inactive. In contrast, mammalian expression systems (e.g., CHO cells, HEK293 cells) are capable of producing recombinant NELL-1 with glycosylation patterns that closely mimic the native human protein, preserving its structure and function (Hasebe et al., 2012). Insect cell-based systems, while previously used for NELL-1 production, yield protein that is relatively unstable and degraded by proteases (Hasebe et al., 2012), and typically introduce simple paucimannosidic glycosylation structures that differ from mammalian glycans (Xu et al., 2024), which could potentially affect bioactivity or introduce immunogenicity (Gupta and Shukla, 2018).

While the specific impact of different glycoforms on NELL-1’s therapeutic efficacy has not been systematically investigated, the choice of expression system is a critical factor in preclinical and clinical development. All recombinant NELL-1 proteins used in the key therapeutic studies discussed in this review (e.g., PEGylated NELL-1) are produced in mammalian cells to ensure proper glycosylation and bioactivity (James et al., 2015; Zhang et al., 2014; Kwak et al., 2015; Tanjaya et al., 2016; James et al., 2016). Future research should focus on defining the precise glycan structures present on human NELL-1 and determining how variations in glycosylation, arising from different production platforms, might influence its pharmacokinetics, receptor binding, and ultimately, its safety and efficacy as a therapeutic agent.

### 2.6 NELL-1 as a tumor suppressor: mechanisms of epigenetic silencing

NELL-1 functions as a tumor suppressor in multiple cancer types through mechanisms involving promoter hypermethylation and subsequent gene silencing. The tumor-suppressive effects of NELL-1 are mediated through inhibition of cell migration, adhesion, and proliferation, as well as potential modulation of Wnt/β-catenin signaling (James et al., 2015; Hooi et al., 2017).

Promoter hypermethylation of NELL-1 has been documented in gastric, colorectal, esophageal, and renal cancers, as well as in lung cancer (Zhai et al., 2019; Gao et al., 2015; Hooi et al., 2017; Radojicic et al., 2012; Wood et al., 2007). This epigenetic alteration leads to downregulated NELL-1 mRNA and protein expression, loss of its tumor-suppressive functions, and consequent promotion of cell proliferation, migration, and invasion (Hooi et al., 2017; Wood et al., 2007).

In gastric cancer, *H. pylori* infection plays a causal role in inducing NELL-1 promoter hypermethylation (Zhang B. G. et al., 2016; Gao et al., 2021). Mechanistically, the *Helicobacter pylori* virulence factor CagA enhances the interaction between PDK1 and AKT, increasing AKT phosphorylation. Phosphorylated AKT subsequently activates NF-κB, which translocates to the nucleus and binds to the DNMT1 promoter, upregulating DNMT1 expression (Zhang B. G. et al., 2016). Additionally, *H. pylori* infection may upregulate DNMT3b through similar pathways (Zhang B. G. et al., 2016). The increased expression of DNA methyltransferases leads to hypermethylation of CpG islands in tumor suppressor gene promoters, including NELL-1 (Zhang B. G. et al., 2016).

Studies have shown that NELL-1 promoter methylation levels are significantly higher in gastric cancer tissues than in normal tissues (Gao et al., 2015; Gao et al., 2021). This hypermethylation correlates with advanced pT stage, pN stage, and poor prognosis, suggesting that NELL-1 methylation status may serve as a prognostic biomarker for gastric cancer patients (Gao et al., 2021).

Why does NELL-1 silencing promote cancer development? NELL-1 exerts its tumor-suppressive effects through multiple mechanisms: (1) inhibition of cell migration and adhesion *via* interactions with integrin β1 and downstream MAPK/ERK signaling (James et al., 2015; Hooi et al., 2017); (2) potential modulation of Wnt/β-catenin signaling, which is frequently hyperactivated in cancers (James et al., 2015); and (3) regulation of the ENO-1-mediated plasminogen activation pathway *via* its ΔE isoform, as discussed in Section 1.2 (Zhao et al., 2018). Loss of NELL-1 expression removes these tumor-suppressive constraints, allowing uncontrolled cell proliferation, enhanced migratory capacity, and increased metastatic potential.

In colon cancer, NELL-1 methylation often co-occurs with aberrant methylation of other tumor suppressor genes such as somatostatin (SST) and tachykinin-1 (TAC1), suggesting a coordinated epigenetic silencing program in colorectal carcinogenesis (Wood et al., 2007). NELL-1 methylation has been identified as a frequent event in primary colon cancers and is associated with downregulated mRNA expression (Wood et al., 2007).

### 2.7 Context-dependent role of NELL-1 in tumor biology

NELL-1 exhibits a remarkable context-dependent role in cancer, functioning as a tumor suppressor in some malignancies while potentially promoting tumor progression in others. This dichotomous behavior—with both increased and decreased expression reported across different tumor types (Zhai et al., 2019; Shen et al., 2015a; Gao et al., 2021; Nakamura et al., 2015)—can be attributed to multiple mechanistic factors, including direct effects on tumor cells and indirect modulation of the tumor microenvironment.

#### 2.7.1 Direct effects on tumor cells

##### 2.7.1.1 Regulation of cell migration through the NELL-1-ΔE/ENO-1 axis

The NELL-1-ΔE isoform plays a distinct role in tumor suppression by binding to enolase-1 (ENO-1) on the cell membrane (Zhao et al., 2018). ENO-1 is a multifunctional enzyme that, in addition to its role in glycolysis, localizes to the cell surface where it acts as a plasminogen receptor. Binding of plasminogen to cell-surface ENO-1 promotes its conversion to plasmin, a protease that degrades extracellular matrix components and facilitates cell migration and invasion (Díaz-Ramos et al., 2012). By binding to ENO-1, NELL-1-ΔE interferes with ENO-1-mediated plasminogen activation, thereby reducing extracellular matrix degradation and cell motility. This mechanism has been implicated in renal cell carcinoma, where NELL-1-ΔE expression is associated with reduced cell migration and adhesion (Zhao et al., 2018; Nakamura et al., 2015).

##### 2.7.1.2 Modulation of cell adhesion and migration via integrin β1 signaling

NELL-1 directly influences tumor cell behavior through interactions with integrin β1 and downstream MAPK/ERK signaling (James et al., 2015). In renal cell carcinoma, NELL-1 inhibits cancer cell migration and adhesion, and its silencing by promoter hypermethylation contributes to tumor progression (Nakamura et al., 2015). Similarly, in gastric and colon cancers, loss of NELL-1 expression removes these tumor-suppressive constraints, allowing uncontrolled cell proliferation and enhanced migratory capacity (Gao et al., 2015; Gao et al., 2021; Mori et al., 2006).

##### 2.7.1.3 Regulation of the extracellular matrix and FAK signaling in osteosarcoma

In osteosarcoma, NELL-1 exhibits a distinct pro-tumorigenic role. NELL-1 is highly expressed and promotes tumor progression by altering the sarcomatous extracellular matrix (ECM) (Yang et al., 2020). NELL-1 expression skews the expression of matricellular proteins associated with increased FAK (focal adhesion kinase) signaling, thereby enhancing cellular invasive potential and metastatic capacity (Bonuccelli et al., 2014; Qin et al., 2023). Genetic deletion of NELL-1 reduces tumor growth, inhibits metastasis, and improves survival in osteosarcoma models (Qin et al., 2023). This context-dependent pro-tumorigenic function may be related to the mesenchymal origin of osteosarcoma and its unique ECM composition.

##### 2.7.1.4 Influence on cancer stem cell differentiation

In lung cancer, NELL-1 overexpression significantly reduces the self-renewal capacity, invasiveness, and chemoresistance of lung cancer stem cells (LCSCs) while promoting their differentiation, by inhibiting the c-MET-Notch signaling pathway (Zhai et al., 2019). This positions NELL-1 as a potential therapeutic agent for targeting this resistant cell population, suggesting a tumor-suppressive role in lung cancer stem cell biology.

#### 2.7.2 Indirect effects via immune modulation

NELL-1 has been shown to modulate immune responses in non-tumor settings, such as promoting anti-inflammatory M2 macrophage polarization in periodontal inflammation (Ni et al., 2024). Whether this immunomodulatory capacity extends to the tumor microenvironment remains to be determined. If NELL-1 similarly promotes M2 polarization in tumors, it could theoretically contribute to an immunosuppressive microenvironment that favors tumor progression. Conversely, NELL-1 may also promote anti-tumor immune responses depending on the context, a possibility that requires direct experimental investigation.

Potential effects on adaptive immunity. Given its expression in lymphoid tissues and modulatory effects on NF-κB and MAPK pathways that are central to lymphocyte activation, it is plausible that NELL-1 may influence T cell or B cell function. However, direct evidence for NELL-1’s role in adaptive anti-tumor immunity remains limited and warrants further investigation. Future studies could explore potential associations between NELL-1 expression and immune cell infiltration in tumors, which may provide insights into its immunomodulatory role in cancer.

#### 2.7.3 Integration of context-dependent effects

The net outcome of NELL-1 signaling in cancer likely depends on the balance between its direct effects on tumor cells (which may be tumor-suppressive in some contexts and pro-tumorigenic in others) and its indirect effects on the tumor microenvironment. Factors determining this balance include: (1) the cellular origin and differentiation state of the tumor (e.g., epithelial *versus* mesenchymal tumors); (2) the epigenetic status of NELL-1 (promoter methylation *versus* active expression); (3) the expression and activity of specific NELL-1 isoforms (full-length *versus* ΔE); (4) the activation status of downstream pathways such as Wnt/β-catenin, MAPK/ERK, and FAK signaling; and (5) the composition of the tumor immune microenvironment.

It is important to note that NELL-1-ΔE represents one of multiple mechanisms through which NELL-1 exerts its tumor-suppressive effects. Other mechanisms include promoter hypermethylation-mediated silencing (Gao et al., 2015; Gao et al., 2021; Mori et al., 2006; Jin et al., 2007), integrin β1 signaling (James et al., 2015), and regulation of cancer stem cell differentiation (Zhai et al., 2019). The relative contribution of each mechanism likely varies depending on the tumor type and cellular context.

Future studies should focus on dissecting these context-dependent mechanisms at the molecular level, particularly the identification of NELL-1-interacting proteins and signaling networks in different tumor types, which will be essential for developing NELL-1-based therapeutic strategies.

The mechanistic switch determining whether NELL-1 functions as a tumor suppressor or exerts pro-tumorigenic effects may depend on multiple factors, including the cellular origin of the tumor (epithelial *versus* mesenchymal), the differential engagement of its receptors (integrin β1 *versus* Cntnap4), and the specific isoforms expressed. For example, in osteosarcoma—a mesenchymal tumor—NELL-1 promotes ECM remodeling and FAK signaling, whereas in epithelial-derived carcinomas (e.g., gastric, lung), NELL-1 acts as a suppressor by inhibiting migration and stemness. Future studies should investigate whether these differences reflect intrinsic properties of the tumor lineage or context-dependent post-translational modifications (e.g., glycosylation patterns) that alter receptor binding affinity.

The seemingly paradoxical role of NELL-1 in different tumors can be partially explained by its engagement of distinct receptors and downstream signaling pathways. In most physiological and tumor-suppressive contexts, NELL-1 binds to integrin β1, leading to the stabilization and nuclear translocation of β-catenin, thereby activating the canonical Wnt/β-catenin pathway (James et al., 2015). This axis promotes differentiation and inhibits proliferation and migration in cells of epithelial origin, such as gastric and lung cancer cells (James et al., 2015; Zhai et al., 2019).

However, NELL-1 can also bind to CNTNAP4, a receptor that is aberrantly expressed in certain mesenchymal tumors like osteosarcoma (Li et al., 2018a; Qin et al., 2023). Engagement of CNTNAP4 by NELL-1 activates the MAPK/ERK signaling cascade, which in these specific contexts drives ECM remodeling, enhances cell motility, and promotes metastasis (Qin et al., 2023). This explains why NELL-1 can exhibit pro-tumorigenic effects in sarcomas while acting as a suppressor in carcinomas. The net outcome of NELL-1 signaling in cancer is therefore determined by a “signaling switch” governed by the relative expression and activation of its receptors, integrin β1 *versus* Cntnap4, and the cellular context in which they are expressed.

#### 2.7.4 NELL-1 in multi-cellular interactions within the tumor microenvironment

One critical aspect of these context-dependent mechanisms that warrants in-depth investigation is the role of NELL-1 in modulating the tumor microenvironment (TME), where it may influence not only tumor cells but also stromal and immune components. Beyond its direct effects on tumor cells and indirect modulation of macrophages, NELL-1 may influence other stromal components of the tumor microenvironment (TME), thereby shaping cancer progression in a context-dependent manner.

Endothelial cells and angiogenesis. NELL-1 has been shown to promote angiogenesis in non-tumor settings *via* the FGF2-AKT-eNOS pathway (Section 3.2.4). In cancer, enhanced angiogenesis could potentially support tumor growth and metastasis. However, whether NELL-1 exerts pro-angiogenic effects within the TME has not been directly investigated. Future studies using tumor xenograft models and endothelial–tumor cell co-cultures are needed to clarify this aspect.

Cancer-associated fibroblasts (CAFs). CAFs are key regulators of extracellular matrix remodeling, drug resistance, and immune exclusion. Given that NELL-1 modulates ECM composition and FAK signaling in osteosarcoma (Qin et al., 2023), it is plausible that NELL-1 may also influence CAF activation or their paracrine signaling to tumor cells. To date, no studies have explored this interaction, representing a significant knowledge gap.

Immune checkpoint molecules. NELL-1 signaling through NF-κB and MAPK pathways could potentially regulate the expression of immune checkpoint molecules such as PD-L1 on tumor cells or immune cells. Given its role in macrophage polarization and the central role of these pathways in immune regulation, NELL-1 may influence the tumor immune microenvironment through multiple mechanisms, a possibility that warrants experimental investigation.

In summary, the role of NELL-1 in the TME extends beyond direct tumor cell modulation, involving potential crosstalk with endothelial cells, CAFs, and the immune checkpoint axis. Unraveling these interactions using advanced models (e.g., organoids, single-cell spatial transcriptomics) will be crucial for understanding the full spectrum of NELL-1’s context-dependent functions in cancer.

## 3 The role of NELL-1 in diseases

NELL-1 functional dysregulation has been associated with the pathogenesis and progression of various tumors. As detailed in Section 1.7, NELL-1 exhibits context-dependent roles in cancer, functioning as a tumor suppressor in some malignancies while promoting tumor progression in others through direct effects on tumor cells (regulating migration, adhesion, ECM interaction, and stem cell differentiation) and indirect modulation of the immune microenvironment. To systematically summarize its expression profiles, relevant signaling pathways, molecular mechanisms, and clinical relevance in tumors, immune-related diseases, and other systemic disorders, a comprehensive overview is presented (Table 1).

**TABLE 1:** NELL-1 expression in diseases.

Category	Disease	Pathway	Mechanism	Clinical sample verified	Cell line/Animal	Experimental methods	Clinical trial	NELL-1 role	References
Tumor	Lung Cancer	NELL-1 inhibits c-MET-Notch signaling	Overexpression of NELL-1 downregulates p-MET, Notch3, and HES1; suppresses self-renewal and invasion of lung cancer stem cells; and enhances chemotherapy sensitivity	no	cell line	Flow cytometry; Western blot; qPCR; soft agar colony formation assay; cell invasion assay	no	Tumor suppressor gene	Zhai et al. (2019)
	Rhabdomyosarcoma	​	High-affinity aptamer targets and inhibits metabolic activity in NELL-1 high-expressing cells	no	cell line	Cell viability/metabolism (MTT); Western blot; flow cytometry	no	Pro-tumor/poor prognosis marker	Duan and Townley (2022)
	Bone Tumors	​	Strong diffuse expression in benign bone-forming and cartilaginous tumors	yes	Patient tissue	Immunohistochemistry (IHC); quantitative RT-PCR	no	Osteogenic differentiation marker	Shen et al. (2015b)
	Osteosarcoma	MAPK/ERK	NELL-1 *via* receptor CNTNAP4 affects cell-ECM interaction, regulates downstream ERK signaling	yes	Cell line, Patient tissue	Transcriptomics; CRISPR/Cas9 knockout; migration/invasion assays	no	Tumor suppressor gene (downregulated)	Qin et al. (2023)
	Gastric Cancer	​	Promoter hypermethylation causes gene silencing; mRNA and protein downregulated	yes	cell line	MALDI-TOF MS (methylation); IHC; PCR	no	Tumor suppressor gene	Gao et al. (2015)
	Renal Cell Carcinoma	​	NELL-1 & NELL-2 downregulated due to promoter hypermethylation; NELL-1 inhibits migration/adhesion	yes	cell line	Quantitative RT-PCR; methylation-specific PCR; migration/adhesion assays	no	Tumor suppressor gene	Nakamura et al. (2015)
	Esophageal Cancer	​	Promoter hypermethylation common; NELL-1/IgG4 co-deposition in kidney suggests potential malignancy	yes	cell line	IHC; methylation analysis	no	Tumor suppressor gene/potential paraneoplastic marker	Zhou et al. (2023)
	Colon Cancer	​	Promoter hypermethylation causes silencing, often co-methylated with SST, TAC1	yes	cell line	cDNA microarray; methylation analysis	no	Potential tumor suppressor gene	Mori et al. (2006)
Non-Tumor	Membranous Nephropathy	​	Specific NELL-1 deposition in GBM co-localizing with IgG; anti-NELL-1 antibodies in serum	yes	cell line	Mass spectrometry; IHC; Western blot	no	Novel autoantigen	Sethi et al. (2020), Wang et al. (2021)
	Craniosynostosis	TGF-β, BMP	NELL-1 overexpression accelerates osteoblast differentiation/mineralization, directly inducing phenotype	yes	Animal, Cell line	Transgenic model analysis; micro-CT; histology/IHC; gene expression (microarray, RT-PCR)	no	Key pathogenic factor	Zhang et al. (2002), Song et al. (2020)
	Autism Spectrum Disorder	NELL-1/Cntnap4 functional axis	NELL-1 is a novel ligand for Cntnap4; haploinsufficiency causes ASD-like behaviors	yes	Animal, Human cohort	Behavioral tests; transcriptomics; GWAS	no	Neurobehavioral regulator	Connolly et al. (2013), Li et al. (2023)
	Osteoporosis	Wnt, Ihh-PTHrP	NELL-1 inactivation disrupts the Ihh-PTHrP pathway by suppressing Gli-1, impairing chondrocyte function and leading to abnormal skeletal development and mineralization	no	Animal	RNA-seq; micro-CT; histomorphometry; Western blot; qPCR	no	Key regulator of bone homeostasis	Chermside-Scabbo et al. (2020), Qi et al. (2019)
	Inflammatory Bowel Disease	​	GWAS shows NELL-1 SNP associated with CD and UC risk	yes	Human cohort	Genome-wide SNP genotyping; association analysis	no	Disease susceptibility gene	Franke et al. (2007)

### 3.1 Tumors

#### 3.1.1 Lung cancer

NELL-1 has been implicated in lung cancer pathogenesis, where its expression is often dysregulated and its function appears context-dependent (Wu et al., 2013). Lung cancer represents the malignant tumor with the highest global incidence and mortality, with smoking being its most important risk factor (Malhotra et al., 2016). However, the genetic profile of lung cancer in non-smokers is distinct. Multiple studies have confirmed that *NELL-1* may function as an essential candidate tumor suppressor gene (Wu et al., 2013).

Beyond its role as a tumor suppressor, NELL-1 exhibits a heterogeneous expression pattern in lung cancer. Analysis from the Human Protein Atlas reveals that NELL-1 protein is detected in a subset of lung squamous cell carcinoma cases, displaying moderate to granular cytoplasmic positivity in cancer cells, suggesting context-dependent functions in different lung cancer subtypes (Uhlén et al., 2015).

Recent investigations have demonstrated that NELL-1 plays a key regulatory role in lung cancer stem cells (LCSCs). It has been reported that its overexpression significantly reduces LCSCs’ self-renewal capacity, invasiveness, and chemoresistance while promoting their differentiation, by inhibiting the c-MET-Notch signaling pathway (Zhai et al., 2019). Given that cancer stem cells are widely recognized as major drivers of tumor recurrence, metastasis, and therapy resistance across multiple malignancies (Haddadin and Sun, 2025), the ability of NELL-1 to induce differentiation of LCSCs positions it as a potential therapeutic agent for targeting this resistant cell population.

Furthermore, emerging evidence links NELL-1 expression to treatment response in lung cancer patients (Sun et al., 2023). Analysis of drug resistance datasets reveals that NELL-1 expression levels correlate with differential responses to both chemotherapy and immunotherapy (Sun et al., 2023). In small cell lung cancer (SCLC), higher NELL-1 expression in malignant cells is associated with chemotherapy resistance (avg_log2FC = 0.445) (Sun et al., 2023). Conversely, in EGFR/ALK mutation-negative non-small cell lung cancer (NSCLC) patients receiving toripalimab combined with chemotherapy, higher NELL-1 expression is associated with better treatment response (avg_log2FC = −0.6477) (Sun et al., 2023). These findings suggest that NELL-1 may serve as a predictive biomarker for immunochemotherapy outcomes in NSCLC patients.

From a clinical perspective, the heterogeneous expression patterns of NELL-1 across lung cancer subtypes and its emerging association with differential treatment responses highlight its potential as both a therapeutic target and a predictive biomarker. These findings warrant further investigation into the prognostic significance of NELL-1 in lung cancer patients. Future studies should focus on elucidating the molecular mechanisms underlying NELL-1’s context-dependent functions and validating its clinical utility in larger, well-annotated patient cohorts.

#### 3.1.2 Rhabdomyosarcoma

NELL-1 has been identified as a potential prognostic marker in rhabdomyosarcoma (RMS), where its overexpression correlates with aggressive tumor behavior (Duan and Townley, 2022). RMS is the most common soft tissue sarcoma in children, characterized by its origin from skeletal muscle precursors and variable clinical aggressiveness (Rubrecht et al., 2025). Beyond known genetic drivers, the molecular mechanisms underlying its metastatic potential are an area of active investigation. In this context, NELL-1 overexpression has been identified in RMS cells, where it promotes metastasis and is associated with poor patient prognosis (Duan and Townley, 2022). This suggests that NELL-1 may play a key role in driving the aggressive behavior of RMS, potentially by regulating critical pro-metastatic pathways. While other molecules such as CD73 have also been implicated in RMS pathogenesis, the relationship between NELL-1 and CD73 in this context remains unexplored, and whether they function within the same or parallel signaling pathways requires further investigation.

#### 3.1.3 Bone tumors and osteosarcoma

NELL-1 exhibits context-dependent expression patterns in bone tumors, functioning as both a differentiation marker in benign lesions and a potential tumor suppressor in osteosarcoma (Shen et al., 2015a; Shen et al., 2015b). Bone tumors represent a rare and heterogeneous group of intraosseous tumors (Choi and Ro, 2021). Current evidence suggests that the bone microenvironment is a key factor in the development, progression, and metastasis of osteosarcoma, a primary bone tumor (Yang et al., 2020). Contributory mechanisms within this niche include oxidative stress induced by reactive mesenchymal stem cells (MSCs),which can induce a metabolic shift toward the Warburg effect (characterized by increased aerobic glycolysis and lactate production), thereby promoting osteosarcoma cell metastasis (Bonuccelli et al., 2014).

Regarding NELL-1 expression, research has confirmed its strong diffuse expression within tumor cells of benign bone-forming tumors, where it is associated with osteogenic differentiation. In contrast, its expression is decreased and more heterogeneous in osteosarcoma, suggesting a context-dependent, bidirectional function in bone tumor biology (Shen et al., 2015a; Shen et al., 2015b).

Moreover, NELL-1/CNTNAP4 signaling axis plays a critical role in osteosarcoma progression. Employing CRISPR/Cas9 to achieve CNTNAP4 knockout resulted in a significant inhibition of tumor growth, osteolysis, and pulmonary metastasis in osteosarcoma models. This phenotype closely mirrored the effects of NELL-1 deficiency, characterized by diminished cellular attachment, migration, and invasive capacity. The genetic ablation of CNTNAP4 was further associated with reduced activity within the MAPK/ERK signaling cascade; notably, the administration of ERK pathway agonists partially rescued the observed functional deficits. At the molecular level, NELL-1 modulates cell-extracellular matrix (ECM) interactions and subsequent ERK signaling through its receptor, CNTNAP4. The clinical significance of this axis is substantiated by human tissue analyses and sequencing data, which confirm the relevance of CNTNAP4 in osteosarcoma pathogenesis. These findings suggest that the NELL-1/CNTNAP4 signaling pathway represents a viable and novel therapeutic strategy for osteosarcoma (Qin et al., 2023).

#### 3.1.4 Gastric cancer

Epigenetic silencing of NELL-1 through promoter hypermethylation has been implicated in gastric carcinogenesis, suggesting its potential as a diagnostic biomarker (Gao et al., 2015). Gastric cancer is the second leading cause of cancer death globally (Sitarz et al., 2018), with complex etiology involving *H. pylori* infection as a major risk factor (Hooi et al., 2017). Epigenetic silencing of NELL-1 through promoter hypermethylation has been implicated in gastric carcinogenesis, suggesting its potential as a diagnostic biomarker (Gao et al., 2015).

As detailed in Section 1.6, *H. pylori* infection induces NELL-1 promoter hypermethylation through a specific molecular mechanism. The *H. pylori* virulence factor CagA enhances PDK1-AKT interaction and increases AKT phosphorylation, which activates NF-κB signaling. Activated NF-κB translocates to the nucleus and upregulates DNMT1 expression, leading to hypermethylation of CpG islands in the NELL-1 promoter (Zhang B. G. et al., 2016).

NELL-1 promoter methylation levels are significantly higher in gastric cancer tissues than in normal tissues, and this hypermethylation correlates with advanced pT (pathological tumor) stage, pN (pathological nodal) stage, and poor prognosis (Gao et al., 2021). Loss of NELL-1 expression removes its tumor-suppressive functions, which are primarily mediated through integrin β1/Wnt/β-catenin signaling rather than the MAPK/ERK pathway (James et al., 2015). Although NELL-1 can activate MAPK/ERK signaling in certain contexts (e.g., osteosarcoma) *via* its receptor CNTNAP4 (Li et al., 2018a; Qin et al., 2023), this pro-tumorigenic effect is not observed in epithelial-derived cancers such as gastric carcinoma. The inhibition of cell migration and adhesion (Rubrecht et al., 2025) is lost upon NELL-1 silencing, thereby promoting gastric carcinogenesis.

These findings indicate that NELL-1 hypermethylation status could serve as a potential early diagnostic biomarker for gastric cancer.

#### 3.1.5 Renal cell carcinoma

NELL-1 is epigenetically silenced in renal cell carcinoma (RCC) through promoter hypermethylation, and its downregulation contributes to tumor progression by promoting cell migration and adhesion (Nakamura et al., 2015). Renal cell carcinoma (RCC) is the most common genitourinary malignancy, with significantly higher incidence in males than females (Bahadoram et al., 2022). While genetic mutations contribute to its pathogenesis, epigenetic regulation plays a key role in RCC development. Functionally, NELL-1 has been found to inhibit cancer cell migration and adhesion, suggesting that its silencing plays an important role in renal cancer progression (Nakamura et al., 2015). These findings highlight the potential tumor-suppressive role of NELL-1 in RCC.

Although multiple pathogenic mechanisms involving molecules such as MFN2, EGFR, and FTO have been implicated in RCC development, their direct relationship with NELL-1 has not been established. Whether NELL-1 interacts with these pathways or functions independently in suppressing RCC progression remains to be elucidated in future studies.

Interestingly, the functional complexity of NELL-1 is further underscored by the discovery of a novel transcript variant, NELL-1-ΔE, which lacks a calcium-binding EGF-like domain and exhibits a specific ability to inhibit cell migration by binding to ENO-1, a function not observed with the full-length protein (Zhao et al., 2018). This suggests that specific NELL-1 isoforms may possess distinct tumor-suppressive activities.

The striking male predominance in RCC incidence raises the question of whether sex differences in NELL-1 expression contribute to this disparity. As noted in Section 1.3, NELL-1 is highly expressed in the testis and prostate, suggesting potential roles in reproductive tissue physiology and possible sex-dependent functions. However, whether NELL-1 expression levels differ between male and female kidneys, and whether such differences influence RCC susceptibility, remains unknown and warrants further investigation.

#### 3.1.6 Esophageal cancer

Esophageal cancer is one of the deadliest cancers worldwide (Domper Arnal et al., 2015), associated with various factors including genetic variants, alcohol, smoking, infections, and gene-environment interactions (Conway et al., 2023), with alcohol and smoking being particularly major risk factors (Wheeler and Reed, 2012). These risk factors, especially alcohol and smoking, drive esophageal carcinogenesis through multiple mechanisms, including chronic inflammation, activation of proto-oncogenes (e.g., RAS), suppression of tumor suppressor genes, and DNA damage; additionally, human papillomavirus (HPV) infection may act synergistically by activating pro-carcinogenic pathways (Radojicic et al., 2012).

As discussed in Section 1.6, NELL-1 functions as a tumor suppressor that is frequently silenced by promoter hypermethylation in multiple cancer types, including esophageal cancer. A case study of a 68-year-old patient with early postoperative recurrence of esophageal squamous cell carcinoma (ESCC) found NELL-1 and IgG4 co-deposition in renal biopsy tissue, with NELL-1 also detected in the cancer tissue. Compared with NELL-1 negative patients, this patient maintained a higher proportion of serum IgG4 even after cancer remission. This finding suggests that the presence of NELL-1, especially with predominant IgG4 co-deposition, in renal biopsies should prompt a high index of suspicion for a concomitant malignancy and justify thorough systemic evaluation (Zhou et al., 2023).

Further research has elucidated the mechanism of NELL-1 inactivation at the epigenetic level, with NELL-1 gene promoter hypermethylation identified as a common event in esophageal adenocarcinoma (EAC). Specifically, no methylation was found in normal esophageal (NE) tissue, but its methylation frequency significantly increased in Barrett’s esophagus (BE), dysplasia (D), and EAC (reaching 41.7%, 52.5%, and 47.8% respectively), and was significantly associated with BE lesion length and decreased patient survival (P = 0.0264), indicating that NELL-1 hypermethylation predicts poor prognosis in early-stage esophageal adenocarcinoma. The study confirmed that demethylating drugs could restore NELL-1 expression, establishing the important pathogenic role of its methylation silencing in EAC progression and suggesting that NELL-1 hypermethylation has the potential to become a biomarker for early diagnosis and prognosis assessment of Barrett’s-associated esophageal cancer (Jin et al., 2007).

Epigenetic silencing of NELL-1 is also implicated in colorectal malignancies.

#### 3.1.7 Colon cancer

Colon cancer ranks among the most common malignant tumors worldwide, with its pathogenesis driven by the aberrant activation of multiple signaling pathways (Hanahan and Weinberg, 2011). At the molecular level, the accumulation of numerous somatic and germline mutations drives dysregulation of key pathways, including Wnt/β-catenin, RAS/RAF/MEK/ERK, PI3K/AKT, and TGF-β, synergistically promoting colon carcinogenesis (Wood et al., 2007). Notably, in addition to classic genetic alterations, epigenetic regulation, particularly DNA methylation, also plays a key role in colon cancer. Further research through genome-wide screening identified NELL-1 as a novel target silenced by promoter hypermethylation in colon cancer; its methylation often co-occurs with aberrant methylation of genes such as somatostatin (SST) and tachykinin-1 (TAC1), suggesting these epigenetic events synergistically contribute to colon cancer pathogenesis. This research confirmed that NELL-1 hypermethylation status was significantly correlated with its downregulated mRNA expression, strongly suggesting NELL-1 may function as a potential tumor suppressor gene. This finding not only deepens the understanding of colon cancer epigenetics but also provides a valuable research direction for developing new diagnostic biomarkers and epigenetic therapeutic strategies (Mori et al., 2006).

### 3.2 NELL-1 in renal and immune-related diseases

#### 3.2.1 NELL-1-associated membranous nephropathy

MN is an autoimmune glomerular disease that begins with podocyte injury and subsequently involves the glomerular basement membrane. Its core pathogenesis involves the deposition of circulating immune complexes and activation of the complement system, leading to tissue damage (Dantas et al., 2023). As detailed in Section 1.5.4, NELL-1 exemplifies this pathogenesis when it becomes a target of autoimmune attack, transitioning from a physiological signaling molecule to a pathogenic autoantigen. Over the past decade, significant progress has been made in elucidating the pathogenesis and improving the clinical management of MN, a common etiology of nephrotic syndrome in adults. Currently, more precise molecular diagnosis can be achieved by identifying specific antigens such as PLA2R, THSD7A, NELL-1, SEMA3B, and dynamic monitoring of circulating autoantibodies provides key guidance for disease assessment and immunosuppressive therapy strategies (Alsharhan and Beck, 2021).

The historical progression of antigen discovery in idiopathic membranous nephropathy (IMN) began with the establishment of the M-type phospholipase A2 receptor (PLA2R) as the primary target antigen (Alsharhan and Beck, 2021), followed by the identification of thrombospondin type 1 domain-containing 7A (THSD7A) as another significant antigen (Tomas et al., 2014). As understanding has advanced, research has evolved to propose an extrarenal antigen exposure model. Within this model, NELL-1 is underscored as a critical antigen responsible for inciting autoimmune injury to podocytes (Liu et al., 2020). Subsequent research has also explored the immune pathogenesis of IMN by constructing miRNA-mRNA regulatory networks, revealing that abnormal NELL-1 expression is closely related to immune regulation, suggesting future research should focus on miRNA-mediated post-transcriptional regulation of NELL-1 (He et al., 2021).

Notably, multiple studies have confirmed NELL-1 as a novel and essential target antigen in PLA2R-negative MN. Its autoantibodies in serum and deposition in kidney tissue are highly specific, marking a distinct primary MN subtype: NELL-1-associated MN (Sethi et al., 2020) (Ahmad and Appel, 2020; Ronco and Debiec, 2021; Lutz, 2021). Subtyping based on podocyte antigens revealed that NELL-1-positive patients constitute approximately 5% of the PLA2R/THSD7A double-negative population (Hanset et al., 2020).

Furthermore, large-scale clinical observations confirm that in addition to anti-PLA2R (70%–80%) and anti-THSD7A (1%–5%) antibodies, approximately 5%–10% of MN cases are associated with anti-NELL-1 antibodies (Stefan et al., 2022; Gu et al., 2021). Specifically, among Chinese primary MN patients, NELL-1 represents a major antigen in the PLA2R/THSD7A-negative population, associated with unique clinicopathological features (Wang et al., 2021).

Traditionally, MN has been classified as primary and secondary. However, with the ongoing discovery of multiple podocyte antigens (e.g., PLA2R in 2009, THSD7A in 2014, and more recently identified NELL-1, EXT1/2, NCAM1, SEMA3B *via* mass spectrometry), this traditional classification system faces significant challenges. Notably, some antigens can be detected in both primary and secondary MN, blurring the distinction between the two categories (Rossi et al., 2022; Fu et al., 2024). Research has demonstrated differences in the positivity rate of NELL-1 between primary and secondary MN, thus improving differential diagnostic accuracy (Iwakura et al., 2022). Based on these findings, there is a growing consensus within the scientific community to adopt molecular subtyping based on target antigens and to advocate for gradually replacing the traditional primary/secondary dichotomy with an antigen-based classification system (Bobart et al., 2021). However, the standardization of antibody detection for minor antigens such as NELL-1 and the complete elucidation of their clinical significance remain subjects for future research and validation (Tesar and Hruskova, 2021).

From a clinical and prognostic perspective, studies have shown that MN associated with the NELL-1 antigen (positivity ∼29%) often presents with relatively mild disease and good prognosis, with most patients potentially not requiring intensive immunosuppressive therapy (Kudose et al., 2021). Besides, cases of dual-antigen-positive MN exist (e.g., PLA2R and NELL-1 coexisting). While their clinical manifestations are similar to those of single-positive patients, they are characterized by higher rates of renal IgG1 deposition and longer durations of clinical remission. Therefore, for PLA2R-positive patients not responding long-term, proactive multi-antigen (including NELL-1, *etc.*) testing is recommended to comprehensively assess etiology and prognosis (Yang et al., 2023). Finally, although rare, NELL-1-associated MN patients may experience severe thrombotic events (e.g., bilateral renal vein thrombosis), necessitating enhanced clinical screening and intervention. Future research should explore the diagnostic value of serum anti-NELL-1 antibodies and optimize anticoagulation strategies for such patients to ultimately improve their prognosis (Kashiv et al., 2024).

Notably, NELL-1’s role in MN extends beyond diagnosis to potential therapeutic implications. As discussed in Section 1.5.4, NELL-1 serves as the target antigen in this autoimmune condition, and therapeutic strategies aimed at inducing immune tolerance to NELL-1 or depleting anti-NELL-1 antibodies could represent novel treatment approaches. Furthermore, the association of NELL-1-positive MN with malignancies (detailed in Section 2.2.6) highlights the importance of comprehensive malignancy screening in these patients, which may lead to early cancer detection and improved outcomes (Wang et al., 2024; Ronco et al., 2021; Hashimoto et al., 2025; Mohamed Jiffry et al., 2023; Fujii et al., 2023; Hu et al., 2024).

The diagnostic utility of NELL-1 in MN, including its sensitivity, specificity, and role in differentiating primary from secondary forms, is discussed in detail in Section 3.1.1, while therapeutic applications are explored in Section 3.3.

#### 3.2.2 Lupus-like membranous nephropathy (LL-MN)

Membranous Lupus Nephritis (MLN) is a unique form of renal involvement in systemic lupus erythematosus, histologically characterized by subepithelial immune-complex deposits in glomeruli (Almaani and Parikh, 2019). However, its histological features often resemble primary MN (pMN), posing diagnostic challenges. Furthermore, a subset of patients termed “Lupus-Like MN” (LL-MN) presents clinically more like pMN than typical SLE, further complicating clinical and pathological diagnosis (Choung et al., 2023). As research advances, the detection of the NELL-1 antigen has emerged as a valuable tool for resolving this diagnostic dilemma. Studies indicate that negative NELL-1 staining helps identify cases of LL-MN that are genuinely associated with systemic autoimmune disease. Conversely, patients presenting with “lupus-like” histological lesions who test positive for NELL-1 are more likely to be classified within the primary MN category. Therefore, NELL-1 immunohistochemistry can effectively assist in distinguishing these two patient groups, which share similar clinical presentations but different etiologies and treatment strategies, and holds significant value for guiding precise clinical treatment (Choung et al., 2023).

#### 3.2.3 Mercury exposure-associated membranous nephropathy

Studies have indicated a clear pathogenic association between mercury exposure and NELL-1-positive MN. The first case report revealed that mercury exposure could trigger NELL-1-positive MN, with the patient’s condition improving after discontinuing mercury-containing medication and receiving anti-proteinuric treatment, suggesting NELL-1 may serve as a novel antigen marker for such disease (Pathak et al., 2022). Subsequent research further confirmed this link, reporting two patients with nephrotic syndrome associated with the use of mercury-containing whitening creams, both of whom were positive for NELL-1 staining in renal tissue; their condition significantly improved after stopping the contaminated source and receiving immunosuppressive therapy. This study emphasizes that NELL-1-positive MN patients with a history of cosmetic product use should be highly suspected for mercury poisoning (Jawandhiya and Gupta, 2024). Larger clinical observations identified intake of traditional or complementary medicines (CAMs) as an essential trigger for mercury exposure and NELL-1-positive MN. Importantly, timely discontinuation and conservative treatment can significantly improve prognosis (Devaraju et al., 2024). Mechanistically, it is now understood that mercury exposure may trigger an autoimmune reaction against NELL-1, leading to MN, thus recommending detailed mercury exposure history investigation for all NELL-1-positive MN patients (Sultan et al., 2023).

Current evidence suggests that NELL-1 is positive in about 50% of mercury-associated MN cases, suggesting its role as an important biomarker for this disease. For PLA2R-negative MN patients, especially those with unclear clinical history, proactive NELL-1 immunohistochemical testing is recommended to improve the identification rate of mercury-associated MN and to avoid misdiagnosis or missed diagnosis due to patients omitting mercury compound exposure history (Srinivas et al., 2024).

#### 3.2.4 Drug-associated membranous nephropathy

Recent clinical reports suggest that certain drugs may be exogenous factors inducing NELL-1-positive MN. For example, a 53-year-old male developed massive proteinuria after 7 months of tiopronin treatment for cystinuria. Renal biopsy confirmed NELL-1-positive MN, with immunohistochemistry showing segmental membranous changes and negative results for common antibodies like anti-PLA2R, suggesting tiopronin may be associated with this type of MN (Santoriello et al., 2023). Similarly, a female patient on long-term alpha-lipoic acid for diabetic peripheral neuropathy also developed NELL-1-associated MN, with significant proteinuria improvement after discontinuing the supplement (Nassar et al., 2023). Besides, a male patient with Wilson’s disease developed nephrotic syndrome after high-dose penicillamine treatment (1200 mg/day). Renal biopsy confirmed MN, while routine serology and PLA2R antibody tests were negative. Despite switching from penicillamine to trientine, proteinuria persisted. Complete remission was achieved only after 21 months of prednisolone combined with mycophenolate mofetil therapy. Given that penicillamine, like bucillamine (known to induce NELL-1 MN), is a thiol-containing drug, retrospective immunohistochemical staining for NELL-1 was performed on the patient’s original renal biopsy tissue, which yielded a positive result, ultimately diagnosing penicillamine-associated NELL-1-positive MN (Dirim et al., 2025). Subsequent research further suggested that in MN patients negative for both PLA2R and THSD7A, the NELL-1-associated subtype should be actively considered. For these patients, a comprehensive examination of potential triggers such as drugs (like the aforementioned thiol compounds) or environmental toxins (like mercury), should be conducted (Priya et al., 2025).

#### 3.2.5 Asbestosis-associated membranous nephropathy

A study reported the case of a male patient with a long history of asbestos exposure. Renal biopsy findings were consistent with MN, and subsequent immunohistochemical staining was positive for NELL-1, indicating a rare association between asbestosis and NELL-1-positive MN. For NELL-1-positive renal disease patients with asbestos exposure history, active screening for occupational lung diseases such as asbestosis is necessary (Sundaram et al., 2025).

#### 3.2.6 Membranous nephropathy with malignancy

In recent years, the incidence of malignancy-associated MN has increased, but its clinical and pathological features remain poorly defined. Studies have indicated that MN typically presents with unique clinicopathological characteristics. For elderly MN patients who are not PLA2R-positive (including those negative for related antigens such as NELL-1), active malignancy screening is recommended. Notably, antigen expression may differ between kidney and tumor tissues, and the specific role of target antigens such as NELL-1 in pathogenesis requires further exploration (Wang et al., 2024).

Mechanistically, the increased cancer risk in patients with glomerular disease may be related to disease-associated immune dysfunction and/or immunosuppressant use. As treatment strategies for secondary glomerular diseases differ from primary ones, inappropriate immunosuppressive therapy may worsen the condition in patients with coexisting cancer. In the differential diagnosis of MN, detecting serum anti-PLA2R/THSD7A autoantibodies and the expression of PLA2R, THSD7A, NELL-1 antigens, and IgG subtypes in renal tissue aids in screening for potential malignancies (Thet et al., 2022).

A key question is whether NELL-1 positivity acts as a causal driver or a prognostic/associative biomarker of malignancy. Current evidence favors the latter interpretation. NELL-1 is expressed in both renal and tumor tissues, and its presence in MN is often associated with concurrent cancer (Wang et al., 2024; Ronco et al., 2021). However, documented cases of NELL-1-positive MN occurring independently of active or recent malignancy suggest that NELL-1 positivity itself is not sufficient to cause cancer (Hashimoto et al., 2025). Instead, it likely serves as a highly specific biomarker indicating a shared antigenic target between the kidney and a (potential) tumor. The detection of anti-NELL-1 antibodies may thus signal an underlying paraneoplastic autoimmune process, making it a valuable indicator for malignancy screening and risk stratification, rather than a direct oncogenic factor.

The high incidence of NELL-1-positive MN in cancer patients can be explained by a paraneoplastic mechanism where the tumor itself is the likely source of antigenic exposure (Wang et al., 2024; Ronco et al., 2021; Hu et al., 2024). In this model, NELL-1 protein may be aberrantly expressed or overexpressed by the malignant cells (Wang et al., 2024; Ronco et al., 2021). This tumor-derived NELL-1 can be recognized by the immune system as “non-self” or “altered self,” leading to a breakdown of immune tolerance and the production of circulating anti-NELL-1 IgG antibodies (Sethi et al., 2020; Wang et al., 2024; Hu et al., 2024). These antibodies then travel to the kidney, where they bind to constitutively expressed NELL-1 on the surface of podocytes (Sethi et al., 2020), forming *in situ* immune complexes and initiating complement-mediated injury characteristic of MN (Dantas et al., 2023). This sequence of events—tumor antigen exposure, systemic autoantibody generation, and targeted attack on renal tissue—constitutes a classic “molecular mimicry” or tumor-antigen-driven autoimmune pathway, as described in other paraneoplastic glomerulopathies (Hu et al., 2024; Thet et al., 2022).

Several factors may contribute to the frequency of this association: (1) the prevalence of NELL-1 expression in certain carcinomas; (2) a cancer-related state of systemic immune dysregulation that favors autoimmunity; and (3) the inherent susceptibility of podocytes, which express NELL-1, to such an attack. Therefore, NELL-1-positive MN in the context of malignancy likely represents a distinct paraneoplastic syndrome where the tumor incites an autoimmune reaction against a shared antigen, with the kidney being a major target organ. This mechanistic understanding underscores why NELL-1 serves as a potent biomarker for occult malignancy and highlights the need for integrated oncology-nephrology care in these patients.

NELL-1, a novel MN antigen, has attracted significant interest due to its association with malignancy. Research indicates that NELL-1 positivity accounts for approximately 8% of primary MN cases, with deposits often segmental and closely related to cancer occurrence, significantly advancing MN molecular subtyping and precision therapy development (Ronco et al., 2021). However, this association is not absolute. A documented case involved an 80-year-old male diagnosed with NELL-1-positive MN after kidney transplant donation, whose remote history of gastric cancer was distant from MN onset, indicating that NELL-1-positive MN in the elderly can occur independently of malignancy (Hashimoto et al., 2025). Further research revealed that, despite independent cases, about one-third of NELL-1-positive MN patients have concurrent malignancies; therefore, long-term close follow-up is warranted for such patients to exclude potential tumors (Mohamed Jiffry et al., 2023; Fujii et al., 2023).

From a therapeutic perspective, the management of malignancy-associated NELL-1-positive MN differs from primary MN. In cases where a tumor is identified, treatment should prioritize oncologic management, as tumor resection has been associated with remission of MN in some cases (Fujii et al., 2023). Immunosuppressive therapy should be approached with caution in patients with active malignancy, as it may promote tumor progression. The role of targeted therapies against NELL-1 or its downstream pathways in this context remains to be explored.

In summary, malignancy-associated MN is an essential type of secondary MN. Recent discoveries of MN-specific antigens such as THSD7A and NELL-1 suggest potential associations with malignancy, but the underlying molecular mechanisms remain unclear. Given the complexity of malignancy mechanisms, future in-depth research into the pathways of these antigens in tumor-associated MN will identify potential targets for developing new therapeutic strategies (Hu et al., 2024).

#### 3.2.7 Pathogenic role *versus* diagnostic biomarker: clarifying the function of NELL-1

The role of NELL-1 varies significantly across the different forms of kidney disease described herein, ranging from a direct pathogenic driver to a secondary biomarker. In NELL-1-associated primary membranous nephropathy (MN) (Section 2.2.1), NELL-1 is not merely a diagnostic marker but the central pathogenic autoantigen. The breakdown of immune tolerance leads to the production of anti-NELL-1 IgG antibodies. These antibodies bind to NELL-1 expressed on podocytes, forming *in situ* immune complexes that activate the complement cascade (e.g., C5b-9), resulting in direct podocyte injury and proteinuria (Sethi et al., 2020; Dantas et al., 2023; Liu et al., 2020). Here, NELL-1 is integral to disease initiation and progression.

In contrast, in MN subtypes associated with exogenous factors such as mercury exposure, certain drugs, asbestosis, or malignancy (Sections 2.2.3-2.2.6), the presence of NELL-1 antigen likely represents a different paradigm. Current evidence suggests that in these contexts, NELL-1 may primarily serve as a sensitive and specific diagnostic biomarker that identifies a distinct histological pattern of injury (Pathak et al., 2022; Jawandhiya and Gupta, 2024; Devaraju et al., 2024; Sultan et al., 2023; Srinivas et al., 2024; Dirim et al., 2025; Priya et al., 2025). Its expression in podocytes could be upregulated in response to various insults (toxic, inflammatory, or paraneoplastic), making it detectable by immune assays. While it is plausible that NELL-1 might modulate local signaling pathways (e.g., NF-κB or integrin-mediated signaling) upon such induction and potentially influence disease severity, conclusive evidence demonstrating its active, causative contribution to disease progression in these secondary forms remains to be established. Therefore, in non-autoimmune-associated MN, the primary clinical utility of NELL-1 detection currently lies in accurate molecular subtyping and diagnosis, with its precise mechanistic role in pathogenesis warranting further investigation.

### 3.3 Craniosynostosis

Craniosynostosis refers to the premature fusion of one or more cranial sutures in infants, a pathological process that limits normal brain growth, leading to abnormal head shape (Kabbani and Raghuveer, 2004). Although various genetic factors associated with craniosynostosis have been discovered, the exact cause of isolated craniosynostosis remains incompletely understood (Arts et al., 2018). Further research has revealed that progenitor cells in prematurely fusing suture tissue have higher intrinsic osteogenic potential compared to those in non-fusing sutures. Concurrently, bone morphogenetic protein 9 (BMP9) can significantly promote osteogenic differentiation in both cell types, upregulate the expression of various osteogenic/osteoclastic regulatory factors, including NELL-1, and induce ectopic bone formation. This suggests that BMP9 may work synergistically with NELL-1 in the pathological process of craniosynostosis (Song et al., 2020). Moreover, mechanistic research using transgenic mouse models specifically overexpressing NELL-1 in the osteoblast lineage confirmed that NELL-1 overexpression can directly induce a craniosynostosis-like phenotype, causing excessive skull growth and premature suture closure. This research further demonstrated that NELL-1 accelerates osteoblast differentiation and mineralization *in vitro*, and its expression is both necessary and sufficient for osteoblast differentiation, thereby revealing the key role of NELL-1 in craniosynostosis pathogenesis (Zhang et al., 2002).

The severity of craniosynostosis correlates with the degree of NELL-1 overexpression, with higher expression levels associated with more extensive suture fusion and greater intracranial pressure (Zhang et al., 2002). While NELL-1’s role in craniosynostosis is primarily pathological, understanding this mechanism has therapeutic implications: modulating NELL-1 activity could potentially prevent or delay suture fusion. However, given NELL-1’s essential roles in normal skeletal development (Section 3.2.1), any therapeutic intervention would require careful temporal and spatial control to avoid adverse effects on overall bone health.

### 3.4 Autism spectrum disorder

Autism Spectrum Disorder (ASD) is a neurodevelopmental disorder where affected individuals typically demonstrate difficulties in social interaction and communication early in childhood, often accompanied by stereotyped repetitive behaviors (Lord et al., 2018). Compelling evidence indicates that genetic factors are the primary risk source for ASD (Bailey et al., 1995). In recent years, research has gradually revealed the potential role of the NELL-1 gene in the neural mechanisms of ASD. A genome-wide association analysis found that the NELL-1 gene was significantly associated with the “fainting, spasms, or syncope” phenotype within ASD endophenotypes, suggesting that this gene may contribute to the neurobiological basis of ASD and provide a potential target for subsequent functional genomic studies (Connolly et al., 2013).

Further research not only supports the above finding but also provides direct evidence for NELL-1 function in the central nervous system through behavioral experiments. This research found that NELL-1 haploinsufficient (NELL-1^+^/^−^) mice exhibit core behavioral deficits reminiscent of autism, including repetitive behaviors and social impairments. This result provides a key mechanistic insight into the frequent co-occurrence of neurobehavioral abnormalities in patients with certain craniofacial syndromes and reveals a novel “bone-brain dialogue” mechanism, thereby opening new research directions for treating related comorbidities (Li et al., 2023).

The relationship between NELL-1 levels and ASD severity appears complex: while haploinsufficiency leads to ASD-like behaviors in mice (Li et al., 2023), the role of NELL-1 in human ASD may involve more nuanced dysregulation. The identification of NELL-1 as a potential ASD risk gene opens possibilities for early diagnostic biomarkers and, potentially, future therapeutic strategies targeting the NELL-1/Cntnap4 signaling pathway (detailed in Section 3.2.6).

### 3.5 Osteoporosis

Osteoporosis is a disease characterized by reduced bone mass and impaired bone microarchitecture, leading to increased bone fragility and fracture risk (Kiel et al., 2007). Research has shown that the occurrence and progression of osteoporosis are significantly influenced by genetic factors, with bone mineral density (BMD) measurement often used clinically for diagnosis and fracture risk assessment (Rivadeneira et al., 2009). Although mechanical loading is an effective exogenous stimulus for bone formation, its osteogenic effect diminishes with age. To investigate this mechanism, one study compared the tibial transcriptomic response to mechanical loading in young (5-month-old) and aged (22-month-old) mice *via* RNA sequencing. Aged mice exhibited significantly fewer differentially expressed genes and weaker pathway activation, including Wnt signaling, extracellular matrix response, and neuromodulatory pathways involving NGF and NELL-1. However, BrdU labeling experiments showed no significant difference in the abundance of osteoblasts between the two groups, suggesting that the reduced bone formation response in aged individuals is primarily due to decreased cellular differentiation capacity and responsiveness of related signaling pathways, rather than insufficient cell proliferation (Chermside-Scabbo et al., 2020). Further molecular mechanism studies confirmed that NELL-1 plays a key role in skeletal development and homeostasis by regulating the Ihh-PTHrP signaling pathway and chondrocyte function. Its gene deficiency leads to phenotypes such as dwarfism and osteoporosis, while exogenous NELL-1 protein supplementation can partially reverse these abnormalities, highlighting its potential value in treating osteoporosis and related skeletal diseases (Qi et al., 2019).

The progressive decline in NELL-1 expression with age directly correlates with osteoporosis severity (James et al., 2015). This mechanistic understanding has been translated into therapeutic strategies: as detailed in Section 3.3.1, NELL-1-based therapies (including PEGylated NELL-1 and combination with PPARγ inhibitors) have shown promise in preclinical osteoporosis models, with the potential to both promote bone formation and inhibit bone resorption through Wnt/β-catenin pathway activation (James et al., 2015; Zhang et al., 2014; Tanjaya et al., 2022; Kwak et al., 2015; Tanjaya et al., 2016; James et al., 2016; Ha et al., 2023; Lee et al., 2015).

### 3.6 Inflammatory bowel disease

Inflammatory Bowel Diseases (IBDs), mainly Crohn’s disease (CD) and ulcerative colitis (UC), are chronic gastrointestinal inflammatory diseases caused by an abnormal immune response to gut microbiota in genetically susceptible individuals (Furey et al., 2019). In genetic mechanism exploration, a genome-wide association study (GWAS) using a multi-stage analysis identified that a single nucleotide polymorphism (SNP rs1793004) in theNELL-1 gene is significantly associated with increased risk for both CD and UC (combined OR = 1.66). This study confirmed NELL-1 as a new susceptibility locus for IBD and validated this association’s reliability in independent samples (Franke et al., 2007). However, this association may vary across populations. A case-control study in a Canadian pediatric and young adult population failed to replicate the previously reported risk association of NELL-1, NCF4, and FAM92B genes with CD from GWAS. This finding suggests that the susceptibility effect of genes such as NELL-1 may be population-specific, and their exact association with IBD needs further validation in broader populations (Amre et al., 2012).

### 3.7 NELL-1 genetic polymorphisms and disease associations

Genome-wide association study (GWAS) data demonstrated that specific single-nucleotide polymorphisms of the NELL-1 gene are associated with osteoporosis, metabolic diseases, neuropsychiatric disorders, neurodegenerative diseases, and specific malignancies (Cheng et al., 2022).

**FIGURE 3:**
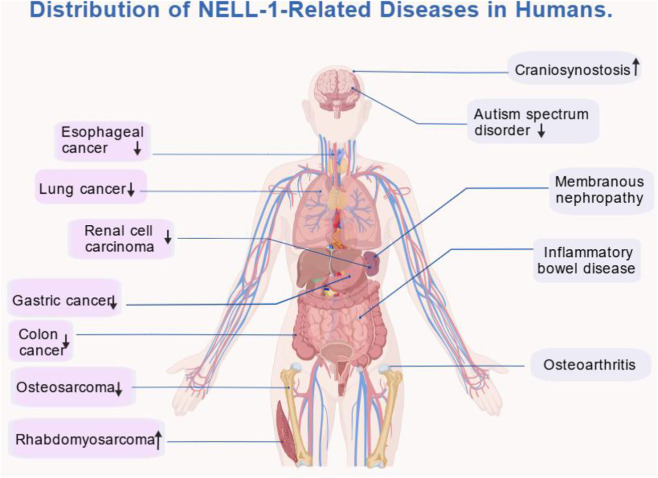
Dysregulation of NELL-1 signaling is closely related to the occurrence and development of multi-system human diseases. Upward arrows (↑) indicate increased expression or hyperfunction; downward arrows (↓) indicate decreased expression or hypofunction. NELL-1 is overexpressed (↑) in craniosynostosis and rhabdomyosarcoma, and downregulated (↓) in autism spectrum disorder and multiple cancers (lung, gastric, renal, esophageal, colon, osteosarcoma). In membranous nephropathy, NELL-1 serves as an autoantigen without altered expression. Genetic polymorphisms link NELL-1 to inflammatory bowel disease and osteoarthritis (no defined expression change). Created with BioGDP.com (Jiang et al., 2025).

Overall, NELL-1 dysfunction plays a significant role in multiple systems, including skeletal, tumor, and immune systems, with its disease spectrum extending far beyond the initial skeletal domain (Figure 3).

## 4 Clinical translation—diagnostics and therapeutics

Building upon the characterization of NELL-1’s disease expression profile and its mechanisms of action, researchers have advanced its translation from basic research to clinical applications. This section systematically reviews NELL-1’s potential as a key molecular biomarker for disease diagnosis (including membrane nephropathy antigens, genetic markers of bone metabolism, and osteogenic and angiogenic factors), as well as its therapeutic strategies and efficacy evaluations for the treatment of osteoporosis, osteoarthritis, periodontal disease, fractures, and other conditions. The review also summarizes relevant mechanisms, experimental approaches, and progress in clinical translation (Table 2).

**TABLE 2:** NELL-1 clinical translation/therapeutics.

Category	Disease/Therapy	Mechanism	Experimental methods	Clinical trial	Animal test	References
biomarker	NELL-1-Assoc. MN	Diagnostic marker for PLA2R-negative MN; detects serum anti-NELL-1 antibodies and glomerular NELL-1 deposition	IHC; Western blot; serum antibody assays	no	no	Wang et al. (2021)
	Bone Metab. Genetic Marker	NELL-1 polymorphisms affect bone quality (trabecular bone score) and predict chemotherapy-induced BMD decline	Human cohort studies; GWAS; SNP genotyping	no	no	Bidkhori et al. (2024), Inaba et al. (2018)
	Lipid Metabolism Regulator	NELL-1 SNPs associated with HCTZ-induced triglyceride increase and n-3 PUFA response	GWAS; SNP analysis; nutritional intervention studies	yes (nutritional)	no	Del-Aguila et al. (2014), Rudkowska et al. (2014)
	Antidepressant Response Predictor	NELL-1 SNP (rs2139423) associated with antidepressant treatment response in MDD	Deep learning models; GWAS dataset analysis	no	no	Lin et al. (2018)
therapy	Osteoporosis	Activates Wnt/β-catenin *via* integrin β1; promotes osteogenesis (Runx2/Osterix ↑) and inhibits resorption (OPG/RANKL modulation); combination with PPARγ inhibitors enhances effects	Knockout models; OVX mouse model; sheep osteoporosis model; PEGylation; BP conjugation	no	Yes (Mouse, rat, sheep)	James et al. (2015), Zhang et al. (2014), Kwak et al. (2015); Tanjaya et al. (2016), James et al. (2016), Tanjaya et al. (2022), Ha et al. (2023), Lee et al. (2015)
	Osteoarthritis	Promotes chondrogenesis *via* Nfatc1-Runx3-Ihh; anti-inflammatory *via* RUNX1 ↑ → NF-κB ↓ → IL-1β/MMP-13 ↓	OA synovial bioinformatics; mouse OA model; histology; IHC; gene expression	no	Yes (Mouse)	Li et al. (2018b), Li et al. (2018c), Li et al. (2020), Zhou et al. (2022), Liu et al. (2022)
	Periodontitis	Promotes M2 macrophage polarization *via* JNK/MAPK; inhibits osteoclastogenesis; modulates oral microbiota	Rat ligature-induced periodontitis; micro-CT; histology; Western blot; ELISA; flow cytometry; RAW 264.7 cells	no	Yes (Rat, mouse)	Ni et al. (2024), Liao et al. (2024)
	Pulpitis	Anti-inflammatory *via* p38/ERK MAPK (IL-6/IL-8 ↓); promotes odontogenic differentiation and angiogenesis	Rat pulp exposure model; human pulp cell culture; ELISA; MAPK inhibitors	no	Yes (Rat)	Cao et al. (2021), Lee et al. (2021), Li et al. (2021), Tian et al. (2019), Wu et al. (2019)
​	Alveolar Bone Defect	NELL-1 gene delivery with 3D-printed scaffold promotes bone regeneration	Rhesus monkey alveolar bone defect model; tissue-engineered bone implant; micro-CT	no	Yes (Rhesus)	Zhang et al. (2018)
	Heart Disease	miR-27a upregulates NELL-1 → inhibits JNK/Wnt/β-catenin → protects mitral valve interstitial cells from TNF-α-induced injury	Human mitral valve interstitial cell culture; transfection; flow cytometry; Western blot	no	no	Chen et al. (2018)
	Fracture	Activates Wnt/β-catenin; synergizes with BMP2; NELL-1_570_ isoform has stronger osteogenic capacity	Mouse fracture model; X-ray; micro-CT; biomechanics	no	Yes (Mouse)	Pang et al. (2015), Shen et al. (2016), Tanjaya et al. (2018), Dobson et al. (2021), Smith et al. (2015)
	Bone Tissue Engineering	Activates MAPK/ERK *via* integrin β1; PEC-based carriers enable co-delivery with BMP-2; chitosan nanoparticles/extended release systems	Rat spinal fusion model; cell culture; ALP activity; qPCR; Western blot	no	Yes (Rat)	Shen et al. (2018), Liu et al. (2019), Wing Moon Lam et al. (2016), Wang et al. (2017), Duarte et al. (2017)

### 4.1 Diagnostic molecular markers

#### 4.1.1 NELL-1-associated membranous nephropathy

Building on the understanding of NELL-1 as a pathogenic autoantigen in membranous nephropathy (MN) detailed in Section 2.2.1, this section focuses on its clinical utility as a diagnostic molecular marker. NELL-1 has been established as a critical diagnostic molecular marker for a distinct subtype of membranous nephropathy (MN). As detailed in Section 2.2.1, NELL-1 serves as the target antigen in approximately 23% of PLA2R-negative MN cases, with high specificity of serum anti-NELL-1 antibodies and granular NELL-1 deposition along the glomerular basement membrane (Sethi et al., 2020; Ahmad and Appel, 2020; Ronco and Debiec, 2021; Lutz, 2021). Among Chinese primary MN patients, NELL-1 constitutes a major antigen in the PLA2R/THSD7A double-negative population (about one-third), supporting the recommendation for routine NELL-1 testing in this subgroup (Wang et al., 2021). Furthermore, NELL-1 immunohistochemistry serves as a valuable tool for differential diagnosis, effectively distinguishing primary MN from lupus-like MN (LL-MN) (Choung et al., 2023). The detection of NELL-1 positivity also serves as a clinical alert for potential underlying conditions, including mercury exposure (Pathak et al., 2022; Jawandhiya and Gupta, 2024; Devaraju et al., 2024; Sultan et al., 2023; Srinivas et al., 2024), drug exposure (particularly thiol-containing compounds such as tiopronin, penicillamine, and alpha-lipoic acid) (Santoriello et al., 2023; Nassar et al., 2023; Dirim et al., 2025; Priya et al., 2025), and occult malignancies (Wang et al., 2024; Ronco et al., 2021; Hashimoto et al., 2025; Mohamed Jiffry et al., 2023; Fujii et al., 2023; Hu et al., 2024). Therefore, NELL-1 functions not only as a diagnostic biomarker for a specific MN subtype but also as a guide for etiological investigation.

#### 4.1.2 Bone metabolism genetic marker

The genetic role of NELL-1 in bone metabolism regulation has gained significant momentum in recent years. A study of about 2,000 elderly Iranians found NELL-1 polymorphisms may significantly affect bone quality (trabecular bone score), providing a basis for NELL-1 as a potential genetic marker for bone metabolic diseases (Bidkhori et al., 2024). Another genome-wide analysis of 363 children with acute lymphoblastic leukemia (ALL) found a specific NELL-1 SNP (rs11025915) that was significantly associated with a sharp decline in lumbar BMD during chemotherapy (*P* = 4.07 × 10^−6^), with a synergistic effect from high dexamethasone levels. This indicates NELL-1 is an essential regulator of chemotherapy-induced bone metabolic abnormalities, and early genetic screening could optimize bone health management (Inaba et al., 2018). Overall, these studies collectively suggest that NELL-1 is a highly valuable genetic marker for bone metabolism.

#### 4.1.3 Lipid metabolism regulator

The function of the NELL-1 gene is not limited to skeletal and dental development; its genetic variants are closely related to lipid metabolism regulation and drug response. Specifically, a genome-wide association study revealed that two SNPs (rs12279250 and rs4319515) in the NELL-1 gene are associated with a significant increase in fasting triglyceride levels in African Americans using the antihypertensive drug hydrochlorothiazide (HCTZ), suggesting NELL-1 may participate in the mechanism of HCTZ-induced metabolic adverse reactions by inhibiting adipogenic differentiation (Del-Aguila et al., 2014). Another study also found that SNPs near the NELL-1 gene are significantly associated with inter-individual differences in plasma triglyceride response to n-3 polyunsaturated fatty acid (PUFA) supplementation; individuals carrying specific risk alleles exhibit a weaker triglyceride-lowering response, further confirming from a nutritional intervention perspective that NELL-1 plays an important role in lipid metabolism regulatory pathways (Rudkowska et al., 2014).

#### 4.1.4 Potential biomarker for predicting antidepressant efficacy

Notably, the importance of the NELL-1 gene extends beyond its classic skeletal and metabolic fields to predict treatment response in mental illness. One study used advanced deep learning models (multilayer feedforward neural networks) to analyze genetic and clinical data, finding that the NELL-1 gene SNP (rs2139423) is significantly associated with treatment response to antidepressants in patients with major depressive disorder (MDD), indicating that NELL-1 has the potential to become a candidate biomarker for predicting antidepressant efficacy (Lin et al., 2018). This finding suggests that NELL-1 genotyping could contribute to personalized psychiatry by identifying patients most likely to benefit from specific antidepressant therapies, though further validation in larger cohorts is warranted.

### 4.2 Biological functions of NELL-1

Beyond its role as a diagnostic marker, NELL-1 exerts diverse biological functions through multiple signaling pathways, regulating key processes including osteogenesis, chondrogenesis, adipogenesis, angiogenesis, inflammation, cranial development, and dental tissue regeneration.

#### 4.2.1 Osteogenic function

Osteogenesis is a complex process involving precise regulation of MSCs by various growth factors (Liao et al., 2022). Interestingly, MSCs have dual potential to differentiate into osteoblasts and chondrocytes, forming the cellular basis for bone repair (Caplan, 1991). Since its discovery in 1999, NELL-1 has been widely confirmed as a key osteogenic factor. By effectively promoting bone formation and regulating bone metabolic balance, NELL-1 demonstrates broad application prospects in treating various skeletal diseases, such as osteoporosis, osteoarthritis, osteonecrosis, and bone defects, and may become an essential future biologic for bone disease treatment (Li and Tian, 2025).

As a multifunctional secreted glycoprotein, NELL-1 exhibits diverse mechanisms of action. It promotes osteogenesis, inhibits adipogenesis and inflammation, plays essential roles in bone tissue engineering and systemic bone disease treatment, and synergizes with BMPs to significantly enhance bone regeneration (Pakvasa et al., 2017). Its molecular mechanisms involve precise regulation of multiple pathways to enhance osteogenesis; molecular optimization, combination therapy, and material-based tissue engineering strategies can further amplify its therapeutic efficacy (Kwak et al., 2013; Yuan et al., 2013). For instance, the Hedgehog agonist SAG combined with NELL-1 synergistically enhanced repair of significant bone defects, significantly promoting new bone formation and improving local vascularization, providing a novel combinatorial strategy for critical-size defects (Lee et al., 2017). Further research confirmed that NELL-1, by activating Wnt/β-catenin signaling, upregulates transcription and the recruitment of Sca-1-positive mesenchymal progenitor cells (MPCs), achieving a 100% spinal fusion rate in a non-human primate model and systemically promoting whole-body bone formation, providing a solid theoretical basis for local and systemic bone regeneration (James et al., 2017). Besides, NELL-1 bidirectionally regulates bone integration by promoting new bone formation *via* regulating the Runx2/Osterix axis and inhibiting osteoclast differentiation by increasing the OPG/RANKL ratio, showing great potential in gene therapy (Lai et al., 2020).

Notably, NELL-1’s biological effects are not static. Research suggests that its isoforms (e.g., full-length vs. truncated) exhibit marked age-dependent differences in their ability to promote MSC proliferation. The NELL-1570 isoform significantly promotes adult mouse MSC proliferation, but this effect diminishes with increasing cell age, revealing isoform-specific and age-related functional differences (Meyers et al., 2019).

In addition, advanced carrier systems have been developed to enable efficient, sustained delivery of NELL-1. Chitosan-stabilized albumin nanoparticles (Chi/NNPs) have been shown to efficiently load NELL-1 and achieve sustained release for up to 8 days while maintaining bioactivity above 82.67%, demonstrating great potential as an efficient delivery system for bone regeneration therapy (Li Y. et al., 2018).

#### 4.2.2 Chondrogenic function

During endochondral ossification, the cartilage matrix serves as a reservoir for potent osteogenic factors, predominantly BMP-7, GDF-5, and NELL-1,Osteoclasts can release these stored factors by resorbing calcified cartilage, synergistically stimulating new bone formation (Iwan et al., 2021). As an osteochondral-specific growth factor, NELL-1, regulated by Runx2, efficiently promotes bone regeneration *via* intramembranous or endochondral ossification. Compared with BMP-2, NELL-1 exhibits greater tissue specificity and does not cause heterotopic ossification. When combined with BMP factors, it demonstrates significant synergy, demonstrating strong clinical translation potential (Zhang et al., 2010).

The regulation of chondrogenesis by NELL-1 involves complex actions at multiple levels and through diverse signaling pathways. Research has confirmed NELL-1 as a key downstream target of transcription factor Runx2 in chondrogenesis; its expression is strictly regulated by Runx2, and it can partially rescue cartilage defects caused by Runx2 deficiency, indicating NELL-1 is a core functional mediator of cartilage generation (Li et al., 2017). Conversely, research also revealed Runx2-independent regulatory mechanisms. One study confirmed NELL-1 regulates chondrogenesis *via* the Runx3-Ihh pathway; even in Runx2 deficiency, NELL-1 can induce Runx3 to activate the Ihh pathway, indicating Runx2’s non-classical function in chondrogenesis (Li et al., 2018c). Further research uncovered a more complete signaling map, revealing NELL-1 regulates chondrogenesis *via* the Nfatc1-Runx3-Ihh signaling cascade, where transcription factor Nfatc1 is the upstream key directly activating Runx3 expression, providing new mechanistic insights and potential therapeutic targets for cartilage regeneration (Li et al., 2018b).

Functionally, NELL-1 not only acts alone but also synergizes with other factors to enhance chondrogenesis. For example, NELL-1 alone or in combination with TGF-β3/BMP-6 significantly accelerates chondrogenic differentiation of human perivascular stem cells (hPSCs), a mechanism related to upregulation of BMP and TGF-β receptors, enhancing cellular response to growth factors (Li et al., 2016).

Finally, in translational research, studies have confirmed that NELL-1 protein, combined with chitosan/hydroxyapatite-tricalcium phosphate (Chi/HA-TCP) composite material, serves as an efficient protein delivery carrier and a bone regeneration scaffold, demonstrating great translational potential (Zhang Y. et al., 2016).

#### 4.2.3 Anti-adipogenic function

NELL-1 is now understood to exert bidirectional regulatory effects on MSC fate. Research has demonstrated that NELL-1 is an effective anti-adipogenic factor, significantly inhibiting adipogenic differentiation of preadipocytes and human adipose-derived stromal cells. Its mechanism may involve activating the Hedgehog pathway (evidenced by upregulation of Ihh, Gli1, Ptc1), providing a new potential strategy for treating diseases such as osteoporosis accompanied by abnormal adipogenesis (James et al., 2011).

NELL-1 promotes osteogenic differentiation in human adipose stem cells (hASCs) through a highly intricate network involving non-coding RNAs and epigenetic modification. Specifically, the pro-osteogenic effect is mediated by lncRNAs that govern the crosstalk between the Hedgehog and Wnt signaling pathways (Xia et al., 2020). Further epigenetic-level research revealed that METTL3 regulates NELL-1-induced osteogenic differentiation of human adipose stem cells by m6A methylating lncRNA RP11-44N12.5, thereby activating the MAPK pathway (Song et al., 2021).

Subsequent research has uncovered a more precise regulatory axis. In this respect, NELL-1 promotes osteogenic differentiation of human adipose stem cells by regulating miR-370-3p and its competing endogenous RNA network, targeting key osteogenic genes such as BMP2 and PTHLH, thereby activating multiple osteogenic pathways (Yu et al., 2020).

**FIGURE 4:**
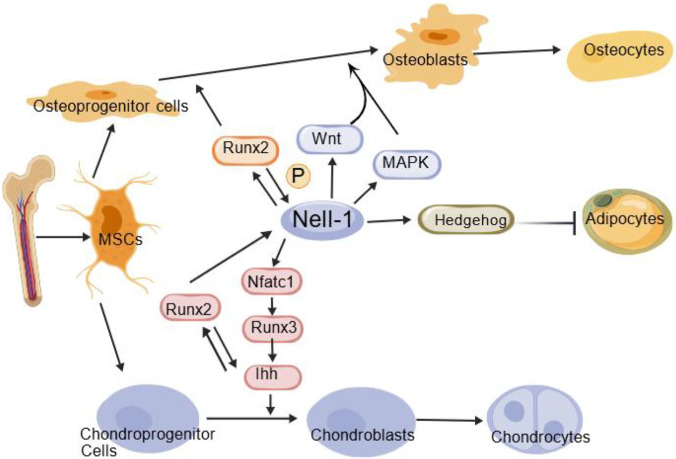
The Role of NELL-1 in Osteogenesis, Chondrogenesis, and Adipogenesis. In mesenchymal cell osteogenic differentiation, NELL-1 mainly activates Wnt and MAPK signaling, promoting osteoblast differentiation. NELL-1 promotes chondrocyte differentiation via the Runx2-NELL-1-Nfatc1-Runx3-Ihh pathway. NELL-1 activates the Hedgehog pathway to inhibit adipogenesis. “P” indicates phosphorylation events involved in signaling cascade activation. Created with BioGDP.com (Jiang et al., 2025).

NELL-1 exhibits multifunctional roles in skeletal and metabolic regulation, particularly in key processes such as osteogenesis, chondrogenesis, and adipogenesis. Its mechanisms involve multiple signaling pathways that collectively form a functional network for tissue homeostasis and regeneration (Figure 4).

#### 4.2.4 Angiogenic function

Research suggests that NELL-1 exhibits significant advantages in promoting cell migration and angiogenesis, with effects superior to BMP2 and requiring lower effective doses. Notably, while NELL-1 does not directly drive osteogenic differentiation, it can optimize the local microenvironment, providing an important alternative strategy for bone defect repair (Fahmy-Garcia et al., 2018). Further research has confirmed that adipose tissue-derived cells combined with NELL-1 yield synergistic effects, combining both pro-angiogenic and osteogenic outcomes (An et al., 2021). Besides, a major study confirmed that human perivascular stem cells isolated from adipose tissue, when combined with NELL-1, synergistically promote osteogenesis and vascularization in a muscle ectopic bone model. Their osteogenic effect was comparable to BMP2, while angiogenic capability was superior, providing an ideal cell/factor combinatorial strategy for vascularized bone regeneration (Askarinam et al., 2013).

#### 4.2.5 Anti-inflammatory function

The multifunctional osteoinductive factor, NELL-1, exhibits significant anti-inflammatory properties that complement its roles in promoting bone and cartilage formation. Mechanistically, NELL-1 has been shown to inhibit overall inflammation and specifically drive the polarization of macrophages away from the pro-inflammatory M1 phenotype toward the anti-inflammatory and tissue-reparative M2 phenotype (Chen et al., 2025). Current evidence suggests that NELL-1 can significantly reduce or completely reverse BMP2-induced local and systemic inflammation (including TNFα, IL6 expression, ROS generation), a mechanism potentially related to NF-κB pathway inhibition (Shen et al., 2013).

#### 4.2.6 Cranial development and neural regulation

Current evidence suggests that Cntnap4 is the functional receptor for the osteogenic protein NELL-1: they bind with high affinity on osteoblast surfaces. Cntnap4 knockout completely abolishes NELL-1-mediated Wnt/MAPK signaling activation and osteogenic effects, and conditional cranial Cntnap4 knockout mice recapitulate the cranial developmental defects caused by NELL-1 deficiency. Besides, the colocalization of NELL-1 and Cntnap4 within human hippocampal neurons suggests the potential formation of a novel ligand-receptor axis in the nervous system, providing a novel perspective on bone-nervous system interaction (Li et al., 2018a). Subsequent research also indicated that NELL-1 is indispensable for cranial neural crest-derived craniofacial skeletal development; its deficiency leads to bone defects and attenuated Wnt/β-catenin signaling. Notably, adult mutant mice exhibit hydrocephalus, suggesting that NELL-1 plays a key role in postnatal cerebrospinal fluid homeostasis regulation (Chen et al., 2020).

#### 4.2.7 Pulp and dentin regeneration

NELL-1, as an important signaling molecule, plays key roles in tooth development, dentin formation, and periodontal tissue regeneration. Current evidence suggests that NELL-1 is widely expressed in the human pulp-dentin complex and can promote pulp cell differentiation toward odontoblasts, thus playing an important role in dentin formation (Liu et al., 2016). Further investigation of its expression pattern revealed that NELL-1 exhibits dynamic spatiotemporal expression characteristics during mouse M development, indicating its important role not only in dentin formation but also in ameloblast differentiation, enamel mineralization, and overall crown morphogenesis (Tang et al., 2013). Notably, NELL-1’s function extends beyond dental hard tissues; it is highly expressed in the dental follicle and, together with Runx2, constitutes a signaling axis crucial for tooth eruption, although their specific regulatory relationship warrants further clarification (Zeng et al., 2022). Besides, in the context of periodontal tissue regeneration, research has confirmed NELL-1 can effectively promote osteogenic differentiation of human periodontal ligament stem cells by upregulating Msx2 (rather than the classic Runx2 pathway), showing great potential in periodontal regenerative gene therapy (Chen et al., 2012). Notably, the osteogenic potential of NELL-1 has been explored for orthodontic applications. In a rat model, localized delivery of exogenous NELL-1 protein was found to accelerate the rate of orthodontic tooth movement, suggesting a promising strategy to reduce overall treatment time. The clinical translation of this approach, however, is complicated by its dose-dependent biphasic effects, which require further investigation (Wang et al., 2018).

The diverse biological functions of NELL-1 across osteogenesis, chondrogenesis, adipogenesis, inflammation, angiogenesis, cranial development, and dental tissue regeneration are summarized in Table 3.

**TABLE 3:** Biological functions of NELL-1.

Function	Signaling pathways/Mechanisms	Key molecules	Functional effects	References
Osteogenesis	Wnt/β-catenin; MAPK/ERK	Integrin β1, Runx2, Osterix, OPG, RANKL	Promotes osteoblast differentiation; inhibits osteoclast activity; enhances bone formation	James et al. (2015), Xia et al. (2020), Huang et al. (2019), Qi et al. (2019), James et al. (2017), Lai et al. (2020)
Chondrogenesis	Nfatc1-Runx3-Ihh; Runx2-dependent and -independent pathways	NFATc1, Runx3, Ihh, Collagen II, Aggrecan	Promotes chondrocyte differentiation; cartilage matrix formation	Zeng et al. (2022), Li et al. (2018b), Li et al. (2018c), Li et al. (2017), Li et al. (2016)
Adipogenesis inhibition	Hedgehog signaling	Ihh, Gli1, Ptc1, PPARγ	Inhibits adipogenic differentiation of MSCs; shifts MSC fate toward osteochondral lineage	James et al. (2015), Pang et al. (2015), Xia et al. (2020), James et al. (2011), Song et al. (2021), Yu et al. (2020)
Anti-inflammation	JNK/MAPK; p38/ERK MAPK; NF-κB	RUNX1, IL-1β, TNF-α, IL-6, CD86, CD206	Promotes M2 macrophage polarization; suppresses pro-inflammatory cytokines; reduces tissue inflammation	Shen et al. (2013), Wang et al. (2018), Ni et al. (2024), Cao et al. (2021), Chen et al. (2025)
Angiogenesis	FGF2-AKT-eNOS	FGF2, AKT, eNOS, VEGF	Enhances endothelial cell migration; promotes new blood vessel formation	Qi et al. (2019), An et al. (2021), Fahmy-Garcia et al. (2018), Askarinam et al. (2013)
Cranial development & Neural regulation	Wnt/β-catenin; MAPK/ERK (*via* Cntnap4)	Cntnap4, Wnt, MAPK	Regulates cranial suture development; involved in neurogenesis and CSF homeostasis	Zhang et al. (2002), Li et al. (2018a), Liu et al. (2016), Chen et al. (2020)
Pulp & Dentin regeneration	Msx2-mediated osteogenesis; p38/ERK MAPK; APR3 interaction	Msx2, Runx2, APR3, DSPP	Promotes odontoblast differentiation; dentin formation; pulp repair; neural differentiation of dental pulp cells	Zeng et al. (2022),Liu et al. (2016), Han et al. (2019), Wang et al. (2018), Tang et al. (2013), Chen et al. (2012)

### 4.3 Therapeutics

**FIGURE 5:**
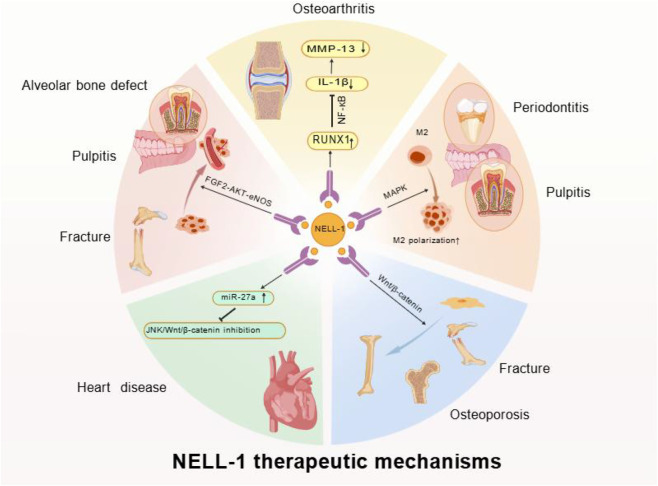
NELL-1 Therapeutic Mechanisms. NELL-1 exerts therapeutic effects through multiple signaling pathways: Wnt/β-catenin: Activates integrin β1 → Runx2/Osterix ↑ (osteogenesis) and OPG/RANKL modulation (anti-resorption) → treats osteoporosis, fracture, bone tissue engineering. MAPK: JNK signaling promotes M2 polarization in periodontitis; p38/ERK suppresses inflammation in pulpitis. RUNX1/IL-1β axis: RUNX1 ↑ inhibits NF-κB → IL-1β/MMP-13 ↓ → chondroprotection in osteoarthritis. FGF2-AKT-eNOS: Enhances angiogenesis → supports repair in dental pulp, alveolar bone defects, fracture. miR-27a: Upregulates miR-27a → inhibits JNK/Wnt/β-catenin → protects against inflammation in heart disease. Created with BioGDP.com (Jiang et al., 2025).

NELL-1 exerts its therapeutic effects through multiple signaling pathways (Figure 5), targeting diverse conditions including osteoporosis, osteoarthritis, periodontitis, pulpitis, heart disease, and bone defects.

#### 4.3.1 Osteoporosis

Building on the understanding that NELL-1 expression declines with age and its deficiency contributes to osteoporosis pathogenesis (Section 2.5) (James et al., 2015), the clinical application of NELL-1 in osteoporosis treatment has demonstrated considerable therapeutic promise, supported by research spanning fundamental mechanisms to advanced delivery strategies. Mechanistically,NELL-1 binds to integrin β1 on the surface of osteoblasts and osteoclasts, activating the Wnt/β-catenin signaling pathway. This leads to β-catenin stabilization and nuclear translocation, resulting in increased expression of osteogenic target genes such as Runx2 and Osterix in osteoblasts, while simultaneously upregulating osteoprotegerin (OPG) and modulating the OPG/RANKL balance to inhibit osteoclast differentiation and activity (James et al., 2015). Consequently, a deficiency in NELL-1 has been implicated in the pathogenesis of age-related osteoporosis (James et al., 2015). Besides, combining NELL-1 with PPARγ inhibitors not only promotes skeletal health but also reduces bone marrow fat accumulation (Tanjaya et al., 2022). To mitigate NELL-1’s rapid *in vivo* degradation, research has shifted toward the development of advanced delivery platforms. A prominent solution involves PEGylation, which effectively prolongs the protein’s plasma half-life and bolsters both its stability and bone-targeting specificity (Zhang et al., 2014) (Kwak et al., 2015). Based on this, intraperitoneal injection of PEGylated NELL-1 (NELL-PEG) has been shown to sustain prolonged systemic exposure and significantly increase bone density in osteoporotic mice (Tanjaya et al., 2016). Further innovation involves conjugating NELL-PEG with the bone-targeting molecule bisphosphonate (BP), constructing BP-NELL-PEG for bone-specific targeting (Ha et al., 2023). In therapeutic validation, recombinant human NELL-1 has demonstrated efficacy in promoting lumbar spine bone regeneration in a large animal (sheep) osteoporosis model (James et al., 2016). This osteogenic potential is further amplified through combination strategies, such as the co-administration of NELL-1 with high-dose human perivascular stem cells, which has been shown to synergistically enhance spinal fusion rates in osteoporotic rats (Lee et al., 2015). In summary, through protein engineering and combination strategies, NELL-1 has demonstrated powerful bone regeneration and bone property improvement efficacy in various osteoporosis models, laying a solid foundation for its clinical translation.

#### 4.3.2 Osteoarthritis

NELL-1 has demonstrated multifaceted potential and value in the treatment of osteoarthritis (OA). Building on the mechanistic understanding of NELL-1’s role in chondrogenesis (Section 3.2.2) and its dysregulation in OA pathology,an initial bioinformatics analysis of OA synovial tissue identified NELL-1 as a core gene, and functional enrichment analysis indicated its key role in immune abnormalities and MAPK signaling, suggesting its potential as a therapeutic target (Zhou et al., 2022). Besides, NELL-1 has been identified as an essential bioactive molecule in MSC-based cartilage repair strategies that enhance cartilage regeneration in large-animal models. However, research in this area currently lacks standardized protocols, necessitating unified systems to improve reproducibility and clinical translation value (Liu et al., 2022). Mechanistically, NELL-1 promotes chondrogenesis by activating the Nfatc1-Runx3-Ihh signaling cascade, leading to upregulation of chondrogenic markers such as Collagen type II and Aggrecan (Li et al., 2018b; Li et al., 2018c). Concurrently, NELL-1 exerts anti-inflammatory effects by upregulating RUNX1 while inhibiting key inflammatory mediators like IL-1β, TNF-α, and subsequent matrix-degrading enzymes (MMP-13, ADAMTS-5) (Li et al., 2020). Crucially, research confirms the dual role of NELL-1 in promoting chondrogenesis and suppressing inflammation. Genetic deficiency of NELL-1 worsens OA pathology and inflammatory responses, whereas its exogenous administration effectively reduces cartilage degradation, joint inflammation, and associated pain. Mechanistically, NELL-1 acts by upregulating RUNX1 while simultaneously inhibiting key inflammatory mediators like IL-1β and subsequent matrix-degrading enzymes, underscoring its strong potential as a disease-modifying OA drug (Li et al., 2020).

In summary, NELL-1 plays a key role in OA: it was identified as a core target *via* bioinformatics from its discovery as a bioinformatics target and functional validation in tissue engineering to its elucidation as a dual-action therapeutic agent, providing solid evidence and development direction for disease-modifying drugs.

#### 4.3.3 Periodontitis

NELL-1 and its engineered complexes exhibit multi-effect mechanisms in immune regulation and tissue repair in periodontitis. Based on the understanding of NELL-1’s anti-inflammatory properties through macrophage modulation (Section 3.2.5), mechanistically, NELL-1 promotes macrophage polarization toward the M2 anti-inflammatory phenotype by activating the JNK/MAPK signaling pathway, leading to increased expression of M2 markers(CD206, Arg-1, IL-10) and decreased expression of M1 markers (CD86, TNF-α, IL-1β), thereby reducing periodontal tissue inflammation and destruction (Ni et al., 2024). In experimental models, NELL-1/gold nanoparticle complexes (NELL-1/AuNPs) are typically administered *via* local injection into the periodontal defect site. This complex exerts a multifaceted regulatory influence, extending beyond the enhancement of macrophage M2 polarization. Its broader therapeutic profile encompasses the significant inhibition of osteoclastogenesis and osteoclastic activity, alongside a beneficial modulation of the oral microbiome. This modulation likely occurs indirectly by ameliorating the inflammatory microenvironment, which in turn alters the ecological niche favoring a healthier microbial composition. Notably, this includes a marked increase in the abundance of beneficial genera such as *Bifidobacterium* (*p* < 0.05). It is important to note that while certain Bifidobacterium species are associated with intestinal health, their role in the oral cavity is complex and context-dependent. The observed increase in this study may reflect a shift towards a less pathogenic microbial community overall, rather than a direct promotion of a single genus linked to caries. By concurrently targeting immunomodulation, bone resorption, and microbial dysbiosis, NELL-1/AuNPs mitigates periodontal tissue destruction through these convergent pathways (Liao et al., 2024).

Current evidence indicates that NELL-1 exerts therapeutic potential through multiple mechanisms, offering new avenues for targeted periodontitis therapy. Regarding biosafety, gold nanoparticles generally exhibit good biocompatibility, but their potential side effects, such as long-term tissue retention or inflammatory responses at high doses, require further evaluation. Thus, its clinical application still requires further evaluation of biosafety and long-term efficacy.

#### 4.3.4 Pulpitis

NELL-1 has multifaceted regulatory roles in the dental pulp field. Mechanistically, NELL-1 attenuates lipopolysaccharide (LPS)-induced inflammation in human dental pulp cells by activating the p38/ERK MAPK pathway, leading to downregulation of pro-inflammatory cytokines (IL-6, IL-8) (Cao et al., 2021). It can serve as a potential target for pulp inflammation therapy (Cao et al., 2021) and promote osteogenic differentiation of odontogenic stem cell spheroids, with significant effects at 10 ng/mL (Lee et al., 2021). NELL-1 also aids in the angiogenic differentiation of dental pulp stem cells (Li et al., 2021) and promotes human dental pulp cell differentiation and mineralization by interacting with APR3 (Tian et al., 2019). Furthermore, NELL-1 combined with BMP2 synergistically promotes reparative dentin formation and reduces inflammation (Wu et al., 2019). Besides, NELL-1 has been shown to drive dental pulp stem cell differentiation toward neural-like cells (Han et al., 2019). In summary, NELL-1 has broad potential in pulp repair and regeneration; however, its clinical translation requires further study.

#### 4.3.5 Alveolar bone defect

Interestingly, a study utilized 3D-printed bioactive glass/chitosan nanoparticle (BD/CSn) composite material loaded with pDNA-NELL-1 and rhesus monkey bone marrow mesenchymal stem cells (BMSCs) to construct tissue-engineered bone. The construct demonstrated excellent osteogenic ability in repairing alveolar bone defects in primates. At 12 weeks post-implantation, the regenerated bone closely approximated native bone in density, hardness, and structure. The NELL-1 gene was confirmed to effectively promote bone regeneration, providing a novel strategy for clinical repair of large-size bone defects (Zhang et al., 2018).

#### 4.3.6 Heart disease

Research has found that miR-27a protects human mitral valve interstitial cells from inflammatory damage by upregulating NELL-1 expression and inhibiting the JNK/Wnt/β-catenin pathway. Importantly, silencing NELL-1 reverses this protective effect, providing a new target for heart valve disease treatment (Chen et al., 2018).

#### 4.3.7 Fracture

NELL-1 has substantial therapeutic potential in bone repair. Mechanistically, NELL-1 activates the Wnt/β-catenin pathway to promote osteogenic differentiation, while simultaneously inhibiting adipogenic differentiation of mesenchymal stem cells (Shen et al., 2016). Studies have shown that systemic injection of NELL-PEG significantly promotes fracture healing in mice, enhancing callus quality and biomechanical strength (Tanjaya et al., 2018). NELL-1 combined with BMP2 synergistically promotes osteogenic differentiation by activating the Wnt pathway and inhibits abnormal adipogenesis (Shen et al., 2016). Its N-terminally truncated isoform NELL-1570 has stronger pro-proliferative and osteogenic capabilities than the full-length isoform (Pang et al., 2015). However, species differences should be noted; e.g., canine MSC osteogenesis highly depends on NELL-1 or BMP-2 activation (Dobson et al., 2021). Overall, NELL-1 has demonstrated excellent clinical application prospects among bone repair biologics (Smith et al., 2015), although its translation must consider limiting factors such as species differences.

#### 4.3.8 Bone tissue engineering

Optimization of NELL-1 delivery systems is crucial for its clinical translation in bone and cartilage regeneration. In bone tissue engineering, NELL-1 exerts its effects by binding to integrin β1 on target cells, activating the MAPK/ERK pathway and subsequently upregulating osteogenic genes (Shen et al., 2018). First, polyelectrolyte complex (PEC)-based carriers can co-deliver NELL-1 with ultra-low-dose BMP-2, significantly increasing spinal fusion rates while reducing side-effect risks (Liu et al., 2019). Furthermore, self-assembling PECs, mediated by heparin, further optimize the controlled release and stability of BMP-2/NELL-1, thus significantly lowering the required growth factor doses (Wing Moon Lam et al., 2016). Concurrently, NELL-1 itself can effectively promote osteogenic differentiation on titanium implant surfaces by activating the MAPK/ERK pathway, thereby enhancing osseointegration (Shen et al., 2018). A dual-delivery system utilizing chitosan nanoparticles and electrospun fibers significantly extends NELL-1 release and sustains its activity, which robustly stimulates the chondrogenic differentiation of human bone marrow mesenchymal stem cells for cartilage regeneration applications (Wang et al., 2017). Finally, review studies have indicated that NELL-1 holds substantial promise as an alternative to BMP-2. By leveraging advanced delivery systems to overcome the critical limitation of uncontrolled burst release, its pathway toward clinical translation is substantially strengthened (Duarte et al., 2017).

NELL-1, combined with novel biomaterial carriers (e.g., PECs, chitosan nanosystems), has made significant progress in co-delivery, dose control, and release kinetics, not only enhancing the efficacy and safety of bone and cartilage regeneration but also providing practical solutions for clinical translation in areas such as spinal fusion and implant integration. Future development relies on further optimization of carrier technology and large-scale clinical validation.

#### 4.3.9 Safety considerations and potential adverse effects of NELL-1-based therapies

Despite the promising therapeutic potential of NELL-1 across multiple diseases, several safety considerations must be addressed before clinical translation.

First, the dual role of NELL-1 as both a therapeutic agent and a pathogenic autoantigen raises important safety concerns. As discussed in Section 2.2.1, NELL-1 is a target antigen in a subset of membranous nephropathy (MN), where circulating anti-NELL-1 antibodies bind to NELL-1 expressed on podocytes, forming immune complexes and activating complement-mediated injury (Sethi et al., 2020; Dantas et al., 2023). Therefore, exogenous administration of NELL-1 protein carries a theoretical risk of inducing or exacerbating NELL-1-associated MN, particularly in susceptible individuals with genetic predisposition or pre-existing autoimmunity. This risk may be influenced by the route of administration (local *versus* systemic), dose, and duration of treatment. Currently, no studies have specifically investigated whether NELL-1-based therapies can trigger *de novo* MN or accelerate disease progression in patients with subclinical anti-NELL-1 autoimmunity. Future clinical trials should include monitoring of renal function, urinalysis for proteinuria, and serial measurement of anti-NELL-1 antibodies in serum.

Second, the potential for off-target effects due to NELL-1’s pleiotropic functions must be considered. NELL-1 is known to play roles in neurogenesis (Wood et al., 2007), chondrogenesis (Mori et al., 2006), and vasculogenesis (Alsharhan and Beck, 2021). Systemic administration of NELL-1 could theoretically affect these processes in unintended ways, leading to neurological abnormalities, ectopic cartilage formation, or aberrant angiogenesis. Preclinical studies in mice with constitutive NELL-1 overexpression have reported a normal lifespan without gross abnormalities beyond the skeleton, suggesting a favorable safety profile (Liu et al., 2016). However, thorough investigation of off-bone effects in relevant animal models is warranted before clinical application.

Third, the immunogenicity of recombinant NELL-1 protein presents a potential hurdle. As a foreign protein, repeated administration of NELL-1 could elicit neutralizing antibodies or hypersensitivity reactions. PEGylation, while improving pharmacokinetic properties, may also reduce immunogenicity, but this requires empirical validation (Zhang et al., 2014; Kwak et al., 2015).

Fourth, the use of NELL-1 in combination with other agents or delivery systems introduces additional safety considerations. For instance, NELL-1/AuNP complexes (Section 3.3.3) utilize gold nanoparticles, which generally exhibit good biocompatibility; however, concerns regarding long-term tissue retention and potential inflammatory responses at high doses necessitate thorough evaluation (Liao et al., 2024). Similarly, viral vectors for NELL-1 gene delivery (Section 3.3.5) carry risks of insertional mutagenesis and immunogenicity (Zhang et al., 2018).

Finally, NELL-1’s tumor-suppressive properties, while potentially beneficial in oncologic contexts (Jin et al., 2007; Zhou et al., 2023), warrant caution in patients with occult malignancies, as modulation of tumor biology could theoretically occur. Long-term carcinogenicity studies in appropriate animal models are needed to exclude any pro-tumorigenic effects.

In summary, while NELL-1-based therapies hold tremendous promise, rigorous preclinical safety assessment and careful patient monitoring in early-phase clinical trials will be essential to identify and mitigate potential adverse effects. Given NELL-1’s role as an autoantigen in membranous nephropathy, particular attention should be paid to renal safety.

### 4.4 NELL-1 as a therapeutic target in oncology

The context-dependent roles of NELL-1 in cancer—ranging from tumor suppression in some malignancies to pro-tumorigenic effects in others—necessitate a precision medicine approach for therapeutic intervention. Based on the mechanistic insights detailed in Sections 1.6–1.7 and the tumor-specific expression patterns summarized in Section 2.1, several actionable strategies can be envisioned.

#### 4.4.1 Strategies for tumors where NELL-1 acts as a suppressor

In cancers where NELL-1 is silenced by promoter hypermethylation (e.g., gastric, colorectal, esophageal, renal, and lung cancers), restoring its expression may re-establish its tumor-suppressive functions. Potential approaches include:

Epigenetic therapy: DNA methyltransferase inhibitors (e.g., 5-azacytidine, decitabine) can reverse NELL-1 promoter hypermethylation, as demonstrated in esophageal adenocarcinoma cells (Jin et al., 2007). Clinical translation would require careful dosing to minimize off-target effects and to ensure selective reactivation of tumor suppressors.

Recombinant NELL-1 protein delivery: Systemic or local administration of NELL-1 protein could suppress cancer stem cell self-renewal and induce differentiation, as shown in lung cancer stem cells (Zhai et al., 2019). However, the short half-life of native NELL-1 necessitates advanced formulations (e.g., PEGylation, nanoparticle encapsulation) to improve pharmacokinetics and tumor targeting.

Gene therapy: Viral (e.g., adeno-associated virus, lentivirus) or non-viral vectors encoding NELL-1 could be employed to achieve sustained local expression within the tumor microenvironment. Preclinical success in bone regeneration (Zhang et al., 2018) suggests feasibility, but tumor-specific promoters and stringent safety controls would be required to avoid ectopic expression.

#### 4.4.2 Strategies for tumors where NELL-1 promotes progression

In malignancies where NELL-1 is overexpressed and contributes to tumor aggressiveness (e.g., osteosarcoma, rhabdomyosarcoma) (Qin et al., 2023; Duan and Townley, 2022), inhibiting NELL-1 signaling represents a rational therapeutic avenue:

Targeting the NELL-1/CNTNAP4 axis: The NELL-1 receptor CNTNAP4 has been shown to mediate pro-metastatic effects in osteosarcoma through MAPK/ERK signaling and ECM remodeling (Qin et al., 2023). Monoclonal antibodies blocking NELL-1-CNTNAP4 interaction, or small molecule inhibitors of downstream effectors (e.g., ERK), could disrupt tumor cell adhesion, migration, and invasion.

Isoform-specific inhibition: Given that the ΔE isoform exerts distinct effects (e.g., binding to ENO-1 and inhibiting migration) (Zhao et al., 2018), therapeutic antibodies or aptamers could be designed to selectively neutralize pro-tumorigenic isoforms while sparing tumor-suppressive ones.

#### 4.4.3 Combination strategies and immunomodulation

NELL-1’s ability to influence the tumor immune microenvironment opens opportunities for combination with immunotherapies:

Macrophage polarization: In non-tumor settings (e.g., periodontitis), NELL-1 has been shown to promote M2-like macrophage polarization (Ni et al., 2024). Although this effect has not been directly demonstrated in the tumor microenvironment, it raises the possibility that NELL-1 could contribute to an immunosuppressive TME by skewing macrophages toward a pro-tumorigenic phenotype. Conversely, blocking NELL-1 might shift macrophages toward an M1-like, anti-tumor state. Combining NELL-1 inhibition with immune checkpoint blockers (e.g., anti-PD-1) could therefore hypothetically enhance antitumor immunity, an idea that warrants experimental investigation.

Angiogenesis modulation: NELL-1 exerts pro-angiogenic effects *via* the FGF2-AKT-eNOS pathway in the context of bone regeneration and vascular cells (An et al., 2021). Whether this translates to enhanced tumor vascularization remains unexplored; however, if confirmed, co-targeting NELL-1 and VEGF pathways might produce synergistic anti-angiogenic effects.

Biomarker-guided patient selection: NELL-1 promoter methylation status, mRNA/protein expression levels, and serum anti-NELL-1 antibody titers could serve as predictive biomarkers to identify patients most likely to benefit from NELL-1-directed therapies. For instance, hypermethylated tumors may respond to demethylating agents, while tumors with high NELL-1 expression might be candidates for receptor-targeted inhibitors.

#### 4.4.4 Safety considerations and future directions

Given NELL-1’s role as an autoantigen in membranous nephropathy (Sethi et al., 2020), any systemic NELL-1-based therapy carries a theoretical risk of inducing or exacerbating autoimmune podocyte injury. Patients enrolled in clinical trials should undergo regular monitoring of proteinuria, renal function, and anti-NELL-1 antibodies. Additionally, in tumors where NELL-1 acts as a suppressor, inadvertent activation of oncogenic pathways (e.g., if the wrong isoform is delivered) must be avoided through rigorous preclinical testing.

Future research should focus on: (i) developing highly specific tools to modulate NELL-1 activity in a context-dependent manner (e.g., isoform-specific antibodies, CRISPR-based epigenetic editors); (ii) validating therapeutic candidates in relevant preclinical models, including patient-derived xenografts and organoids that recapitulate the TME; and (iii) establishing robust biomarker panels to guide patient stratification. The convergence of these efforts will be essential to translate the complex biology of NELL-1 into effective, safe oncologic therapies.

## 5 Summary

This article systematically reviews the molecular structure and biological characteristics of NELL-1, its expression and function in various diseases, and its progress towards clinical translation in diagnostics and treatment. First, NELL-1 plays a central role in skeletal development, immune regulation, and neural function through multiple signaling pathways. Second, it demonstrates significant pathological and therapeutic relevance in tumors, membranous nephropathy, osteoporosis, craniosynostosis, and other diseases. Finally, important advances have been made in developing NELL-1-based diagnostic markers and tissue engineering strategies, highlighting its broad clinical potential.

Future research should further focus on NELL-1’s isoform-specific functions, regulatory networks within tissue microenvironments, and the exploration of shared mechanistic pathways across different diseases. Simultaneously, leveraging cutting-edge technologies will be crucial for deeper mechanistic investigation and clinical translation. For instance, gene editing tools (e.g., receptor-specific CRISPR screening or base-editing) can precisely dissect the functional contributions of NELL-1 and its putative receptors in disease-specific contexts; kidney and tumor organoid models offer unique platforms to study NELL-1 antigenicity in membranous nephropathy and its matrix-dependent effects on tumor behavior within a human-relevant microenvironment; and integrating spatial multi-omics with artificial intelligence can map the expression of NELL-1 and its receptors to specific cellular outcomes and pathological features, uncovering novel regulatory circuits and predictive biomarkers.

Notably, several challenges and knowledge gaps remain to be addressed to fully realize NELL-1’s translational potential. Key hurdles include the precise identification and validation of its high-affinity receptors across different tissues, a clearer understanding of how its roles depend on context (and are sometimes opposing) in various diseases, and the optimization of delivery systems for sustained bioactivity and tissue-specific targeting *in vivo*. Furthermore, the interplay between NELL-1 isoforms and their distinct functions, as well as the long-term safety and immunogenicity of NELL-1-based biologics, require comprehensive evaluation in advanced preclinical models.

The convergence of these approaches is expected to accelerate the translation of NELL-1 from basic research to clinical precision medicine, ultimately providing new therapeutic strategies and diagnostic tools for multi-system diseases, including skeletal, immune, neural, and neoplastic disorders.
